# Pharmacometabolomics Detects Unreported Clopidogrel Metabolites in the Urine of Kidney and Liver Transplant Recipients

**DOI:** 10.3390/metabo16030210

**Published:** 2026-03-21

**Authors:** Cassandra Piccolotto, Stephan J. L. Bakker, Vincent E. de Meijer, Gérard Hopfgartner, Peter Fodran, Frank Klont, TransplantLines Investigators

**Affiliations:** 1 Unit of Pharmaco Therapy, Epidemiology & Economics, Groningen Research Institute of Pharmacy, University of Groningen, Antonius Deusinglaan 1, 9713 AV Groningen, The Netherlands; 2 Division of Nephrology, Department of Internal Medicine, University Medical Center Groningen, University of Groningen, Hanzeplein 1, 9713 GZ Groningen, The Netherlands; s.j.l.bakker@umcg.nl; 3 Division of Hepatobiliary Surgery and Liver Transplantation, Department of Surgery, University Medical Center Groningen, University of Groningen, Hanzeplein 1, 9713 GZ Groningen, The Netherlands; v.e.de.meijer@umcg.nl; 4 Life Sciences Mass Spectrometry, Department of Inorganic and Analytical Chemistry, University of Geneva, Quai Ernest Ansermet 30, 1211 Genève, Switzerland; gerard.hopfgartner@unige.ch; 5 Department of Chemical and Pharmaceutical Biology, Groningen Research Institute of Pharmacy, University of Groningen, Antonius Deusinglaan 1, 9713 AV Groningen, The Netherlands; p.fodran@rug.nl; 6 Department of Clinical Pharmacy and Pharmacology, University Medical Center Groningen, University of Groningen, Hanzeplein 1, 9713 GZ Groningen, The Netherlands; 7 Group of Authors on Behalf of the Transplant Lines Biobank and Cohort Study, University Medical Center Groningen, University of Groningen, Hanzeplein 1, 9713 GZ Groningen, The Netherlands; transplantlines@umcg.nl

**Keywords:** clopidogrel, drug metabolism, human, liquid chromatography, mass spectrometry, metabolomics, pharmacometabolomics, SWATH, transplantation

## Abstract

**Background/Objectives**: Clopidogrel is a widely prescribed antiplatelet prodrug that requires bioactivation, primarily by the polymorphic CYP2C19 enzyme. Genetic variation in this enzyme leads to differences in active metabolite formation and has prompted the development of pharmacogenetics-guided prescribing. However, current pharmacogenetic strategies are grounded in drug metabolism knowledge derived from mass balance studies conducted in small groups of healthy volunteers. This narrow evidence base may limit the data’s applicability to real-world settings, where factors like polypharmacy or altered organ function may influence drug response. **Methods**: Pharmacogenetics could benefit from real-world drug metabolism and excretion studies, which we conducted for clopidogrel in 38 kidney and 16 liver transplant recipients from the TransplantLines Biobank and Cohort Study (NCT03272841), utilizing existing LC-SWATH/MS pharmacometabolomic data. Clopidogrel-associated metabolic signals were identified using xenobiotic metabolism knowledge and literature-reported pathways. **Results**: Across both transplant groups, 26 clopidogrel-associated features were prioritized, of which some matched previously reported urinary metabolites, had previously been observed in plasma, or represented previously unreported metabolites. Clopidogrel carboxylic acid predominated in kidney transplant recipients, whereas its glucuronide form was most abundant in liver transplant recipients. Notably, unmetabolized clopidogrel was consistently detected across all patients. Moreover, our data support a thiol desulfurization route, aligning with emerging evidence of clopidogrel’s role as a hydrogen sulfide-releasing drug. **Conclusions**: More (putative) clopidogrel metabolites were detected than previously reported, demonstrating the value of pharmacometabolomics in expanding our understanding of drug metabolism. This approach provides novel data that may complement pharmacogenetics research to understand clopidogrel response variability among treated patients.

## 1. Introduction

Personalized medicine is reshaping healthcare by tailoring disease prevention, diagnosis, and treatment methods to the unique biological, lifestyle, and genetic characteristics of individuals. This concept deviates from a more traditional “one-size-fits-all” approach and places the patient at the center of care. The field has furthermore gained momentum with advancements in “omics” domains, particularly since the launch of the Human Genome Project in 1990 [1].

Pharmacogenetics (PGx) has emerged as a key field in personalized medicine, exploring how genetic characteristics affect drug response. This field holds the potential for improving patient outcomes and reducing unnecessary drug use, by informing drug therapy and dosage selection. By integrating pharmacology with genomics, PGx aims to optimize treatment efficacy and minimize adverse effects linked to interindividual genetic variability [2,3], reportedly explaining 20–40% of variability in drug response [4,5]. Much of this variation arises from genetic differences in drug transporters and metabolizing enzymes, particularly those from the cytochrome P450 (CYP) superfamily, which governs the activation, deactivation, and/or elimination of many medications [3,6].

Among CYP superfamily members, the polymorphic CYP2C19 isoenzyme is responsible for metabolizing a wide range of therapeutics, including antidepressants, proton pump inhibitors, and antiplatelet agents [7,8]. To date, CYP2C19 has over 35 allelic variants listed in the online repository of the PharmVar Consortium (at the time of writing the manuscript, in the winter of 2025–2026) [9], many of which give rise to a wide variety of metabolizer phenotypes that are associated with clinically relevant variability in drug response [7,8,10]. Notably, 20–30% of individuals in European populations were recently predicted to be CYP2C19 ultrarapid metabolizers compared to 2% in East Asian populations. Conversely, only around 3% in European populations were poor metabolizers compared to 14% in East Asian populations [11]. Such genetic variation likely results in different pharmacokinetics from those assumed and elucidated during drug development, which form an important basis of their dosing regimens [12].

Clopidogrel is an antiplatelet prodrug prescribed to prevent cardiovascular events, and a key example of a CYP2C19 substrate [13,14,15]. Individuals with reduced functioning alleles have been associated with decreased formation of clopidogrel’s active metabolite, leading to reduced drug efficacy [16,17,18,19]. Meanwhile (ultra)rapid metabolizers can exhibit an increased drug response and may be at an increased risk of bleeding due to stronger antiplatelet effects [20,21,22,23]. The corresponding genetic insights have informed PGx-based prescribing guidelines, such as those developed by the Clinical Pharmacogenetics Implementation Consortium (CPIC) in 2011, with further refinements made in 2013 and 2022 [24,25,26].

Despite these genotype-based recommendations, ensuing clinical studies have produced mixed results. Notably, several studies found no significant differences in platelet reactivity or cardiovascular outcomes between different CYP2C19 metabolizer phenotypes [27,28,29]. These findings may link to the fact that clopidogrel metabolism is more complex than CYP2C19-mediated activation alone. In fact, approximately 85% of an administered dose is inactivated by the carboxylesterase 1 (CES1) enzyme, leaving only 15% available for bioactivation [30,31]. Of that remaining fraction, a portion is further inactivated by paraoxonase-1 (PON1), generating an endo-thiol metabolite with no antiplatelet activity which adds to the incompleteness of clopidogrel’s bioactivation [32,33].

These metabolic data suggest that pharmacogenetics may need to broaden its focus beyond CYP2C19 alone when optimizing clopidogrel therapy. It also seems warranted to re-examine clopidogrel’s metabolism, particularly because much of the current knowledge still relies on a radiolabeled mass-balance study conducted in the 1990s [31]. Notably, that study failed to identify clopidogrel’s active thiol metabolite (H4) [31,34]. Furthermore, this Belgium-based study included only six healthy male volunteers (aged 24–32 years), making it unlikely to be representative of clopidogrel’s intended patient population.

Here we take the first step to re-examine clopidogrel’s metabolism by compiling existing metabolism knowledge through a literature review and by conducting a urinary drug metabolite elucidation study in real-world clopidogrel users. For this, we utilized pharmacometabolomics (PMx), a variant of metabolomics based on liquid chromatography (LC) coupled to high-resolution (HR) mass spectrometry (MS). PMx allows for the identification of both expected and previously unreported metabolites, and it has recently been shown to enable real-world drug metabolism studies [35] and was proposed as a tool to complement PGx [36]. Moreover, we focused on kidney (KTR) and liver transplant recipients (LTR), as they represent clinically relevant populations in which clopidogrel is frequently prescribed for cardiovascular risk management and where (patho)physiological alterations, comorbidities, and polypharmacy are likely to influence its metabolism.

## 2. Materials and Methods

### 2.1. Literature Review of Clopidogrel Metabolism

A literature review was conducted using PubMed and Embase to identify articles related to the metabolism or bioactivation of clopidogrel. An overview of the search strategy is provided in the Supplementary Material (Method S1). Titles and abstracts were screened for relevant keywords, and full texts were subsequently retrieved to assess eligibility based on predefined exclusion criteria (see Figure S1 for PRISMA flow diagram). Only peer-reviewed research articles published in English were considered. Exclusion criteria included studies focused on drug–drug interactions, analytical method development for clopidogrel analysis, or topics unrelated to clopidogrel metabolism.

### 2.2. Clinical Samples and LC-MS Profiling

This study used existing clinical and 24 h urine PMx data from kidney and liver transplant recipients from the TransplantLines Biobank and Cohort Study (NCT03272841), which was approved by the Institutional Review Board of the University Medical Center Groningen (UMCG; decision METc 2014/077 on 25 August 2014) and was conducted in accordance with UMCG Biobank Regulations, the Declaration of Helsinki, and the Declaration of Istanbul [37]. Regarding the clinical data, these were collected during study visits and included body mass index (BMI), estimated glomerular filtration rate (eGFR; calculated using the CKD-EPI creatinine equation 2021), serum albumin, and serum alanine transaminase (ALT). Additional clinical information, including age, sex (determined based on the Dutch Personal Records Database), transplantation details, and self-reported medication usage was obtained from participant medical records [38]. Clopidogrel use was the only exception to self-reported medication and was instead based upon compound identification (through spectral library matching) in the PMx dataset (see Figure S2). This approach was informed by pilot work, which indicated that clopidogrel in urine could be relevant for assigning exposure statuses. Additionally, we used a commercial software tool (SCIEX PeakView, version 2.2.0.11391) and a commercial spectral library (SCIEX ‘Forensic’, version 1.1) under vendor-recommended settings, considering accurate mass and isotope patterns at the MS1 level and spectral similarity at the MS2 level. Potential false positives were identified by manually reviewing all positive hits, whereas potential false negatives were assessed by manually inspecting data from self-reported clopidogrel users and by performing a feature-based check. The latter consisted of a *t*-test group comparison between positive and negative hits, specifically aimed at identifying potentially high signals among exposure-negative samples for the feature corresponding to clopidogrel, while also assessing the features putatively corresponding to clopidogrel’s main carboxylic acid and carboxylic acid glucuronide metabolites. For samples suspected to represent false negatives, spectral library matching results were also manually evaluated, and exposure statuses were updated where applicable.

Regarding the PMx analyses, we separately studied the four cohorts according to transplant type and study design: KTR cross-sectional (≥12 months post-transplantation), KTR longitudinal (3, 12, and 24 months post-transplantation), LTR cross-sectional (≥12 months post-transplantation), and LTR longitudinal (pre-transplantation and/or 3, 6, 12, and/or 24 months post-transplantation). All analyses were furthermore performed in an untargeted, data-independent acquisition (DIA) mode using reversed-phase LC coupled to time-of-flight MS, operated in the ‘SWATH’ mode, as initially described by Hopfgartner et al. [39] and Gillet et al. [40]. More specifically, LC separation was performed over a 12 min gradient of 5–80% solvent B (methanol) in solvent A (5 mM ammonium formate and 0.1% formic acid in water), and the MS method had a cycle time of 1 s during which one MS1 scan (*m*/*z* 100–1250) and 38 MS2 scans (*m*/*z* 40–1250) with variable Q1 precursor isolation windows were acquired. Urine samples were collected following a standardized 24 h collection protocol (developed internally for generic biobanking purposes, thus lacking metabolomics-specific pre-analytical procedures) and were stored at −80 °C after aliquoting. Prior to measurement, samples were thawed, vortex-mixed, and centrifuged, and an internal standard mixture (50 pmol/10 µL) was added to 50 µL of the supernatant, after which 24 µL (≡20 microliters of urine, 20 pmol per internal standard) of the resulting mixture was analyzed by LC-MS. A detailed description of sample preparation procedures, the LC–MS parameters, batch design, and quality control procedures has been published elsewhere [35], and the PMx data have previously been deposited in an open-access repository (https://doi.org/10.26037/yareta:64ruex2sxff5nenyfyexurzs3m).

### 2.3. Feature Selection

Three-dimensional features (i.e., mass-to-charge ratio (*m*/*z*), retention time, peak area) were extracted and aligned from mass spectrometry data using SCIEX MarkerView software (version 1.3.1), with the settings shown in Table S1 and without application of a distinct feature normalization procedure. Next, feature data were analyzed separately for KTR and LTR in both cross-sectional and longitudinal datasets, notably extracting statistically significant features (MarkerView’s *t*-test module; α = 0.05, with Bonferroni correction) with the PMx-based clopidogrel exposure status as the grouping variable (step 1: statistics-based filtering). Low-abundance features, defined as having a median relative abundance below 1.0% of the most abundant feature, were excluded from downstream analysis (step 2: mathematical, relative abundance threshold-based filtering). Furthermore, features meeting the significance threshold were manually filtered to remove isotopes, in-source fragments, and adducts (e.g., sodium, potassium) (step 3: manual, pattern-based filtering). However, if a feature met the abundance threshold in at least one subgroup, it was kept for further evaluation (step 4: mathematical, rule-based inclusion). Lastly, the resulting lists of prioritized features were manually assessed using SCIEX PeakView software (version 2.2.0.11391) for potential misalignment or signal merging caused by automatic feature detection (step 5: manual, visual inspection-based inclusion/exclusion). In this process, fragment spectra were also screened for characteristic chlorine isotope patterns, specifically assessing mass shifts of +1.997 Da to confirm associations as clopidogrel metabolites.

### 2.4. Metabolite Identification (As “Putatively Characterized Compound Classes”)

Clopidogrel-use-associated features identified in clopidogrel-positive samples were further investigated following manual signal integration to generate quantitative values due to potential signal merging during automatic feature detection. This manual integration was performed using SCIEX MultiQuant software (version 2.1), applying a ±5 mDa extraction window, a 2.0-point Gaussian smoothing width, and a retention time tolerance of 30 s for the (default) peak finding algorithm, which integrates peaks based on a valley-to-valley principle. Integrations were subsequently reviewed individually, ensuring that both valleys were at baseline level, as was the case for most signals. In the case of peak splitting, integrations were adjusted by applying a perpendicular drop to the baseline from the valley between the adjacent peaks. Notably, the sum of all adjacent integrations equaled the area of a combined integration from the left-baseline valley of the first peak to the right-baseline valley of the last peak. Next, for each prioritized feature, MS1-level precursor ion peaks were integrated to enable relative comparison, and MS2-level residual precursor and fragment peaks were integrated for qualitative feature identification purposes (see Table S2). The relative abundance was then calculated for each prioritized metabolite by dividing the (MS1-level) signal intensity of each individual substance by the sum of the signal intensities of all the substances found per drug user. Accordingly, the relative abundances for each clopidogrel-positive sample were normalized such that the summed signal intensities of all detected metabolites totaled 100%, analogous to reporting approaches used in human mass balance studies conducted during drug development that quantify the relative distribution of drug metabolites excreted by clinical trial participants.

Initial metabolite identifications were guided by established metabolic pathways reported in the literature. Additionally, features were evaluated based on human xenobiotic metabolism knowledge while considering the chemical properties of clopidogrel (e.g., molecular weight, molecular formula, known fragment spectra) and the principles of the LC-MS analytical workflow employed (e.g., reversed-phase LC, positive electrospray ionization, collision-induced dissociation). Discrepant assignments were subsequently reviewed by additional co-authors, and final identifications were reached by consensus. To explore inter-individual variability and group-level metabolic patterns, principal component analysis (PCA) was performed using relative abundance data. The PCA was conducted using the SCIEX MarkerView software with Pareto scaling and aimed to identify clustering amongst clopidogrel positive samples and detect potential outliers.

## 3. Results

### 3.1. Clopidogrel Metabolism in the Literature

Clopidogrel was historically detected through phenotypic (ADP-induced platelet aggregation) screening and was developed further, despite limited initial understanding of the enzymes, metabolites, and pathways involved in its metabolism [41]. The metabolism and excretion knowledge available was furthermore largely based on the radiolabeled mass balance study conducted by clopidogrel’s developers, in which 6 healthy males aged 24 to 32 were given a single oral dose of 75 mg ^14^C-labeled clopidogrel and, four weeks later, oral administrations of 75 mg (unlabeled) clopidogrel for seven consecutive days [31]. In this study, the drug was found to be rapidly absorbed and extensively metabolized by the liver, with the inactive clopidogrel carboxylic acid (CCA) metabolite constituting the majority of the absorbed radioactivity [30,31].

For the remaining, not inactivated fraction of clopidogrel, it was reported to undergo oxidative metabolism via the CYP enzymes to form a pharmacologically active metabolite [42]. CYPs were initially described in the literature as key mediators in this bioactivation through a two-step pathway, with several isoforms having been implicated in this process, including CYP3A4, CYP2C19, CYP1A2 and CYP2B6 [15,43,44,45,46]. Among these CYPs, CYP2C19 has consistently been highlighted in the literature for both its mechanistic role in forming the active metabolite and its clinical importance [15,45]. It is currently also the only CYP enzyme included in guidelines for pharmacogenetic testing, as recommended by the CPIC [26]. This recommendation aims to ensure sufficient enzymatic activity for the oxidative conversion of 2-oxoclopidogrel into a sulfenic acid intermediate, which subsequently forms the active thiol metabolite [43,47]. Additionally, one study indicated that glutathione significantly facilitates this conversion through the transient formation of a glutathione conjugate that may potentially lead to downstream metabolic complexity [48]. Regardless of the exact metabolic pathway, the active thiol metabolite produced has proven difficult to detect and characterize due to its transient nature and high reactivity. In fact, it took several years to accurately characterize the active thiol metabolite and confirm its biological activity. This metabolite was found to be short-lived in circulation making it difficult to trap, with a pharmacokinetic study reporting a half-life of approximately 45 min [49]. In 2000, 3 years after approval, four thiol isomers (H1–H4) were eventually resolved using human liver microsomes [34,50]. Of these metabolites, only the isomer with an S-configuration at C7 and the Z-configuration at the C3–C16 double bond (named thiol H4) exhibited antiplatelet activity, by irreversibly binding to the P2Y Purinoceptor 12 (P2Y12) receptor via a disulfide bond [34,50,51].

**Figure 1:**
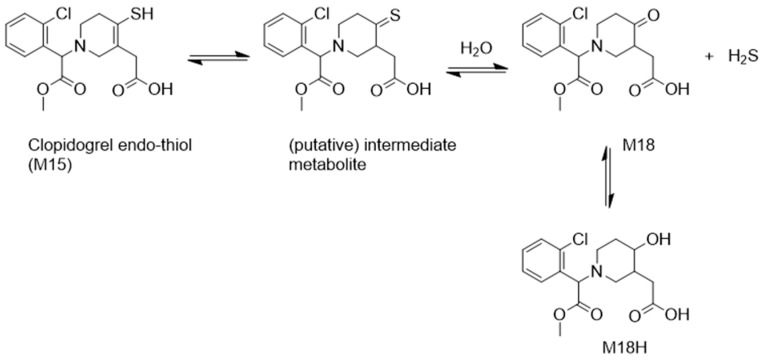
Potential endo-thiol metabolism pathway for H_2_S release. Metabolites M18 and M18H have both been identified in human plasma during in vivo studies [54].

Initially, the formation of this active metabolite was thought to be mediated by both the CYPs and the less commonly studied PON1 enzyme [52]. Regarding the latter, one study emphasized the role of PON1 genotypes in influencing clopidogrel efficacy, suggesting that genetic variability in this enzyme might account for interindividual differences in response [52]. However, subsequent research clarified that PON1 does not generate the active thiol metabolite, but, instead, converts 2-oxoclopidogrel into an inactive endo-thiol metabolite [32,45,53]. Interestingly, this pathway has since drawn attention due to its potential role in hydrogen sulfide (H_2_S) donation. Specifically, research on H_2_S donating drugs has led to speculation that clopidogrel might exert biological effects via H_2_S release, possibly through a PON1-mediated pathway [54]. In this regard, a 2018 study in mice and six healthy human volunteers proposed the presence of both M18 and M18H in plasma (see Figure 1), suggesting that the endo-thiol metabolite may undergo a tautomeric shift, enabling thione-to-ketone conversion for H_2_S release [54].

The endo-thiol pathway is not the only rather uncommon metabolic pathway observed for clopidogrel, given findings of in vitro studies utilizing human liver cell lysates in which clopidogrel transesterification occurred in the presence of ethanol, forming ethyl ester metabolites [55,56]. This pathway may be reminiscent of the formation of cocaethylene from simultaneous ingestion of ethanol and cocaine, with ethanol inhibiting CES1, the primary esterase responsible for clopidogrel hydrolysis [55,56,57]. Such ethylated clopidogrel metabolites may potentially be observed in individuals who consume alcohol, illustrating how external exposures, such as alcohol use, can alter metabolism in ways not always captured by controlled trials (in healthy volunteers).

Altogether, several clopidogrel metabolites may plausibly be detected in urine, including the inactive CCA metabolite and its glucuronide (commonly referred to as CAG), as well as M18H, which is the most likely to appear in urine as the downstream product of the PON1 pathway. Additionally, M18 itself and potentially also some transesterified metabolites could be encountered, while pharmacologically active thiol metabolites, such as thiol H4, are unlikely to be observed due to their reactivity and instability.

### 3.2. Characteristics of Kidney and Liver Transplant Recipients

For clopidogrel metabolite identification, PMx data from 24 h urine samples were taken from four different cohorts. One cohort is a cross-sectional study of 570 KTR sampled at least 12 months post-transplantation where PMx confirmed 12 clopidogrel positive samples, all matching with the self-reported drug use information. The other KTR cohort is a longitudinal study of 163 recipients who were all sampled at 3, 12, and 24 months post-transplantation, where 26 samples tested clopidogrel positive, of which 24 were expected to be positive based on self-reported drug use. Similarly, the cross-sectional study of 316 LTR sampled at least 12 months post-transplantation identified seven clopidogrel positive samples by PMx, all matching with the self-reported drug use information. The last cohort is an LTR longitudinal study of 176 recipients who were all sampled either pre-transplantation and/or at 3, 6, 12, and/or 24 months post-transplantation, where nine samples were clopidogrel positive, of which eight were expected to be positive from self-reported data.

Among the clopidogrel users across the cohorts there is a similar age range, BMI and sex distribution of females, with the exception of the longitudinal KTR cohort where 65% of the samples belonged to females (see Table 1). Meanwhile, the eGFR, serum albumin levels and serum ALT levels are more similar within the KTR and LTR subgroups. Notably, the KTR have eGFR values around 50 mL/min/1.73 m^2^ which are consistent with decreased kidney functioning associated with chronic kidney disease (eGFR < 60 mL/min/1.73 m^2^ for ≥3 months) [58]. The time since transplantation varies greatly amongst all cohorts due to their differential study designs; however, it is important to note that the clinical parameters may be skewed for the last cohort as four of the nine samples in the longitudinal LTR study are pre-transplant samples.

### 3.3. Selection of Clopidogrel-Use-Associated Features

**Figure 2:**
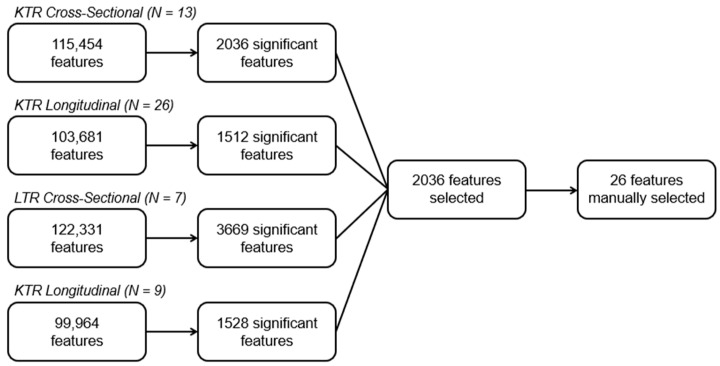
Schematic overview of feature selection in kidney and liver transplant cohorts. Statistical significance was determined using a *t*-test (α = 0.05, with application of Bonferroni correction). Features were then filtered as described in Section 2.3 and manually assessed to identify potential misalignment or signal merging introduced by automatic feature detection.

The LC-MS analyses yielded 115,454 and 103,681 features in the KTR groups and 122,331 and 99,964 features in the LTR groups (see Figure 2). Once the significant features were filtered to remove isotopes, adducts, in-source fragments, and low abundance features, 37 features remained for consideration (see Table 2). A manual data assessment was deemed necessary, as clopidogrel is a chiral and rather hypermetabolized compound and the reversed-phase liquid chromatography utilized in this study is not well suited for baseline-resolution of chiral or structurally similar isomers. Also, for this reason, the retention time domain was not used to differentiate features, and instead, one representative median signal intensity per *m*/*z* was selected. This manual inspection eventually led to the exclusion of 11 features, which represent features that had been merged with a closely eluting compound by the automated feature extraction software, but where signal intensities were too low across all datasets to justify further consideration.

The final set of 26 prioritized features, including some present at trace levels, were detected across all cohorts and their relative abundances are presented in Table 3. The retention times listed in the table reflect the peak apexes that stood out during manual inspection; however, the true number of underlying peaks may be higher due to co-elution of structurally related compounds. To generate the quantitative values shown in Table 3, peak areas from MS1 extracted ion chromatograms were manually integrated. Where appropriate, closely eluting peaks were collectively summed to allow for the capture of closely eluting signals, particularly for chiral or structurally similar metabolites.

### 3.4. Metabolite Identification (As “Putatively Characterized Compound Classes”)

**Figure 3:**
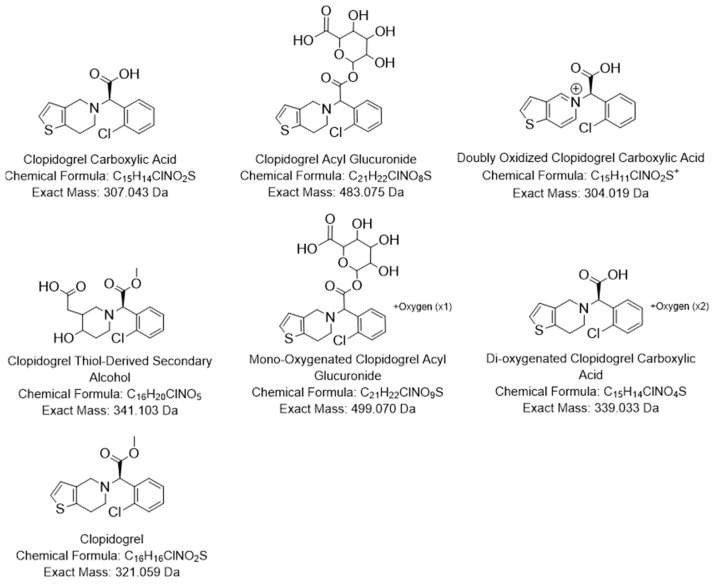
Putative structural formulas of the 7 most abundant clopidogrel metabolites based on this study’s findings.

Manual inspection and integration of clopidogrel-positive urine samples using the *m*/*z* values of the 26 prioritized features yielded 51 distinct signals associated with clopidogrel exposure, each corresponding to a (confidently) separate peak apex at a unique retention time (Table 4, Figures S3–S28). Given the large number of prioritized features, identification efforts were focused on the most abundant features, being those showing relative abundances above 1% in two or more cohorts, following joint consideration of peaks with matching *m*/*z* values. This resulted in 7 features, all representing “putatively characterized compound classes” in accordance with level 3 identification criteria outlined by the Metabolomic Standards Initiative (MSI), which could be identified [59]. Their putative structural formulas, including exact mass and chemical formula, are presented in Figure 3. Among the 7 compounds, one feature corresponded to clopidogrel itself, which had previously been identified via spectral library matching (see Figure S2), thus actually representing a level 2 identification, or “putatively annotated compound”, according to the MSI. Although clopidogrel was detected only in minor quantities across the other three cohorts, it notably reached a relative abundance of 10.3% in the cross-sectional KTR cohort.

The two major metabolites we identified were CCA and its phase II glucuronide conjugate (CAG), consistent with findings from previous mass balance studies [31,60]. These metabolites were identified based on their monoisotopic mass as well as the expected mass differences and lower retention times relative to clopidogrel. CCA, the phase I metabolite formed via CES1 hydrolysis, was present at higher median relative abundances in KTR (26.6% and 33.6%) compared to LTR (19.3% and 25.1%). In contrast, CAG, a phase II conjugate of CCA, was more abundant in LTR samples (38.5% and 33.6%) relative to KTR (29.6% and 27.2%). This distinction in metabolic profiles thus resulted in a different most abundant metabolite depending on type of transplant received.

Furthermore, we detected 4 additional metabolites in the urine of KTR and LTR that, to our knowledge, have not previously been reported in this matrix. These included a di-oxygenated CCA (*m*/*z* 340.04), a mono-oxygenated CAG (*m*/*z* 500.08), a doubly oxidized CCA (M10; *m*/*z* 304.02), and a clopidogrel-thiol-derived secondary alcohol (M18H; *m*/*z* 342.11). Further, metabolic annotations were based on monoisotopic mass and characteristic mass differences from known precursor molecules, and most matched with previously published literature, with the exception of the previously unreported signals at *m*/*z* 340.04 and *m*/*z* 500.08. The M10 metabolite has been described in human liver microsome studies characterizing CYP-mediated metabolism. This metabolite is described as a result of thiophene metabolism and the latter arising from CYP3A4/5 mediated piperidine ring metabolism [61]. Additionally, Zhu and Zhou also identified a signal at *m*/*z* 306.03 as a oxidized CCA, which is of interest as it may align with one of our minor metabolites detected at that same signal (see Table 4) [61]. In contrast, M18H is consistent with the prior reported in vivo finding of this metabolite as a downstream metabolite of H_2_S release in plasma [54].

The relative abundance of metabolite M18H showed intra-cohort study consistency, with little variation between transplant types (Table 4). Among the phase I metabolites, *m*/*z* 340.04 and *m*/*z* 304.02 were consistently abundant across study groups, except for the lower levels in the longitudinal LTR cohort. The sole unreported phase II metabolite, *m*/*z* 500.08, was more abundant in KTR at 3.9% and 4.5%, compared to 2.6% and 3.4% in LTR.

In addition to the more confidently identified compounds, several low abundance features (relative abundance < 1.0% across the cohorts) exhibited mass difference patterns indicative of well-known metabolic transformations. For instance, feature *m*/*z* 326.06 was linked to another feature, *m*/*z* 445.06, differing by 119.0041 Da, with the fragment spectrum of the latter showing characteristic losses of 121.020 Da relative to the residual precursor, consistent with cysteine conjugation. Additional related signals included *m*/*z* 445.06 and 459.07 (+14.0157 Da), 445.06 and 461.05 (+15.9949 Da), and 461.05 and 637.08 (+176.0321 Da), all of which align with known chemical modifications such as methylation, mono-oxygenation, and glucuronidation, respectively. While these features were not prioritized due to their low abundance, the observed mass relationships support a tentative inclusion in the clopidogrel metabolic pathway.

### 3.5. Metabolite Profile Patterns and Variability

**Figure 4:**
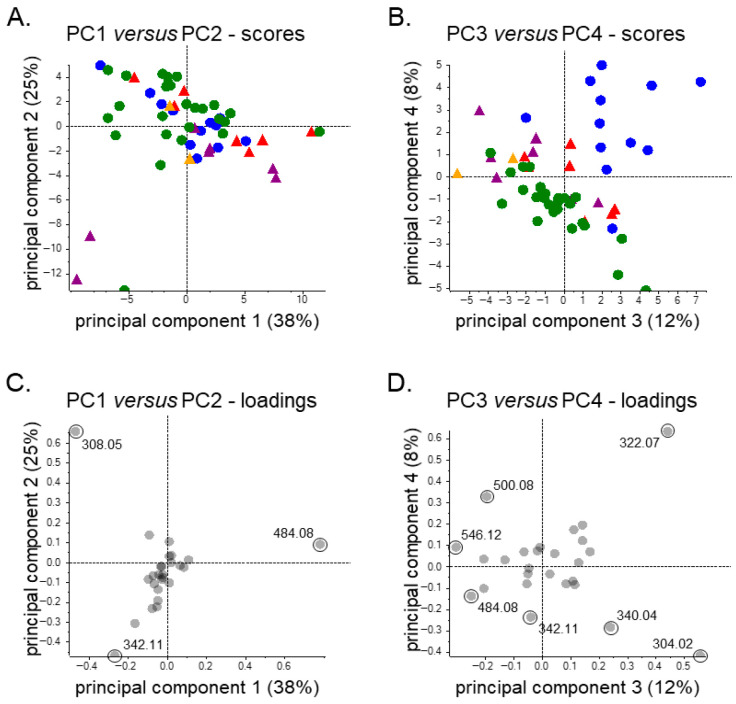
Pareto-scaled (**A**,**B**) scores and (**C**,**D**) loadings plots for unsupervised principal component (PC) analysis of relative urinary clopidogrel metabolite data of kidney transplant recipients participating in the cross-sectional (N = 13; blue dots) or longitudinal (N = 26; green dots) sub-cohorts of the TransplantLines Biobank and Cohort Study, liver transplant recipients participating in the cross-sectional (N = 7; red triangles) or longitudinal (N = 7; purple triangles) sub-cohorts of this study, and individuals who belong to the latter sub-cohort but who were still awaiting liver transplantation surgery (N = 2; orange triangles). This figure includes all PCs showing a percentage of variance explained of at least 5%. The substances highlighted in the bottom panes are labeled using the *m*/*z* values presented in Table 4.

Unsupervised PCA was conducted to explore patterns of variability in the urinary clopidogrel metabolite profiles across subgroups for potential hypothesis generation purposes that may inspire future research projects, using the relative data summarized in Table 4. The first two principal components (PC1 and PC2) explained the majority of variance in the data, accounting for 38% and 25%, respectively, while PC3 and PC4 contributed an additional 12% and 8% (Figure 4A,B). Within PC1, the primary contributors were clopidogrel’s main metabolites, CCA and CAG, whereas PC2 reflected differences in M18H versus CCA and CAG, which were mostly driven by three samples. Some separation by transplant type is furthermore evident along PC1, as KTR tended to associate more strongly with CCA, while the LTR clustered toward CAG (Figure 4A), thus mirroring the relative abundance patterns described in Table 4. For PC2, the three samples appearing as outliers in the lower left quadrant of the PCA plot were characterized by reduced CAG and CCA levels and an elevated signal for M18H. When the analytical (e.g., internal standard signals) and clinical data (e.g., comedication, kidney function, liver markers) of these samples were considered, it yielded no relevant insights for this metabolite pattern, although it should be noted that well-designed and -powered (pharmacoepidemiologic) studies would be needed to properly assess potential associations with such clinical features. Lastly, further separation was evident along PC3 and PC4 and showed clear differentiation of the KTR cross-sectional subgroup from the others, mostly driven by higher relative abundance values for the clopidogrel prodrug itself (Figure 4B).

A subgroup of individuals with rather favorable values for key pharmacokinetic parameters were selected for further comparison via PCA. Although not clinically classified as healthy (and representing a very small sample), these individuals were considered to have characteristics supportive of typical drug metabolism and elimination. Specifically, the inclusion criteria for this subgroup were a body mass index between 19 and 30 kg/m^2^, an estimated glomerular filtration rate of at least 60 mL/min/1.73 m^2^, serum albumin levels between 35 and 50 g/L, and serum alanine aminotransferase levels below 34 U/L. This resulted in 3 samples within the cross-sectional KTR, 4 samples in the cross-sectional LTR and longitudinal KTR, and no longitudinal LTR samples.

**Figure 5:**
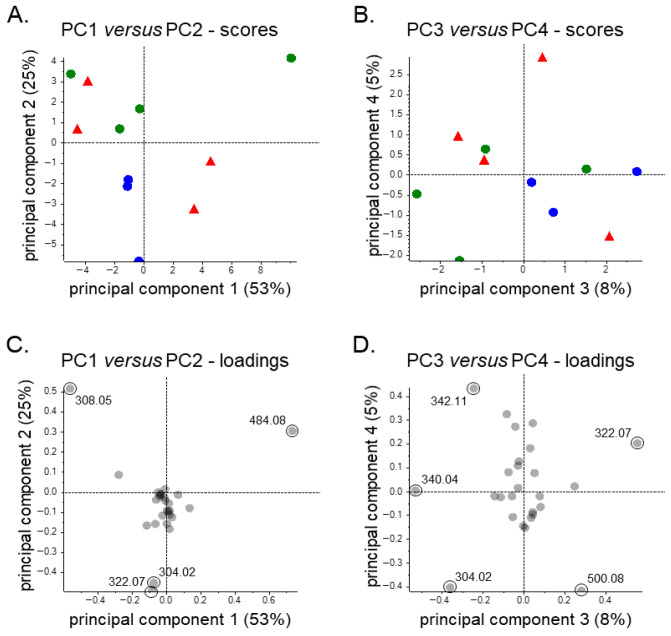
Pareto-scaled (**A**,**B**) scores and (**C**,**D**) loadings plots for unsupervised principal component (PC) analysis of relative urinary clopidogrel metabolite data of selected kidney transplant recipients participating in the cross-sectional (N = 3; blue dots) or longitudinal (N = 4; green dots) sub-cohorts of the TransplantLines Biobank and Cohort Study and selected liver transplant recipients participating in the cross-sectional (N = 4; red triangles) sub-cohort of this study. Regarding this selection, included individuals had a body mass index between 19 and 30, estimated glomerular filtration rate of at least 60 mL/min/1.73 m^2^, serum albumin levels between 35 and 50 g/L, and serum alanine aminotransferase levels below 34 U/L. This figure includes all PCs showing a percentage of variance explained of at least 5%. The substances highlighted in the bottom panes are labeled using the *m*/*z* values presented in Table 4.

The resulting PCA plots (Figure 5) showed that PC1 accounted for a substantial proportion of the variance (53%), while PC2 accounted for 25%, with PC3 and PC4 contributing considerably less. PC1 retained its primary contributors, CCA and CAG, and KTR continued to cluster driven by CCA abundance values. Among cross-sectional KTR, alignment with the clopidogrel signal (*m*/*z* 322.07) was still observed, though now primarily along PC2 instead of PC3. The latter can be explained by the absence of the three outlier samples from Figure 4 in this subgroup analysis.

## 4. Discussion

This study investigated real-world metabolism and urinary excretion of clopidogrel in kidney and liver transplant recipients using existing PMx data. These data indicated that most of the expected urinary metabolites of clopidogrel were detected, including the main inactive metabolites CCA and CAG, along with clopidogrel itself and various minor metabolite signals [30,31,60]. This study did not find/prioritize several known metabolites, including the thiol H1, H2, H3, and H4 metabolites, the structurally related endo-thiol metabolite, and their precursor metabolite 2-oxoclopidogrel, which are presumably formed transiently, are highly reactive, and/or are known to be low abundance species. Additionally, metabolite profiles were found to be different between the KTR and LTR cohorts, notably showing higher relative abundances of the inactive CCA metabolite in KTR, while its glucuronidated version, CAG was the most abundant in LTR.

In agreement with the initial mass balance study completed in healthy volunteers [30,31], the most abundant urinary metabolite peaks identified were CCA and CAG [60]. However, the relative abundance differed somewhat by transplant type. CCA was more abundant in KTR, with median levels approximately 7–8 percentage points higher than in LTR, whereas CAG was more abundant in LTR by about 6–9 percentage points (Table 4). One possible contributor to this discrepancy is the mono-oxygenated clopidogrel acyl glucuronide (*m*/*z* 500.08), which showed slightly higher levels in KTR (approximately 1 percentage point higher), suggesting increased phase I metabolism in KTR compared to LTR. Still, the magnitude of this difference implies other mechanisms are likely involved. Reduced renal clearance among KTR may also play a role, potentially limiting the urinary excretion of CAG. In this regard, the major excretion route for various other (acyl) glucuronides is through the kidneys, and their excretion may be sensitive to reductions in glomerular filtration rate [62,63,64]. As the KTR have an approximately 30% lower eGFR compared to LTR, this could partly explain their lower urinary CAG levels. Alternatively, other factors may contribute to variation in glucuronidation rates between groups, including various genetic and non-genetic factors (e.g., comedication, dietary substances, lifestyle exposures) which were not captured in the available clinical dataset.

Beyond detection of the well-known urinary metabolites, the identification of the M18H metabolite adds to emerging evidence that clopidogrel may function as a H_2_S donor. Previously reported only in plasma, its presence in urine may support the possibility of an alternative bioactivation pathway for clopidogrel going beyond its use in cardiovascular medicine [54]. Interestingly, the three prominent outliers observed in PCA all showed elevated levels of M18H alongside reduced levels of CCA and CAG. Their profiles may suggest reduced CES1 activity or increased conversion of clopidogrel to 2-oxo-clopidogrel, leading to increased fractions of its downstream metabolites. Regarding the former, a known CES1 low-functioning variant (i.e., rs71647871), while rare given a reported 3.7% prevalence in Caucasian populations, has been linked to increased concentrations of clopidogrel’s active metabolite and enhanced platelet inhibition [65,66,67,68]. Reduced CES1 activity may also arise from enzyme inhibition caused by drug–drug or drug–excipient interactions. The latter has been recently demonstrated by Nijdam et al., with the Kolliphor^®^ EL excipient that is present in cyclosporine A (CsA) capsules, which led to reduced mycophenolate mofetil conversion to its active metabolite, mycophenolic acid [69]. Diminished CES1 function could allow more clopidogrel to be diverted toward CYP-mediated bioactivation and thus also PON1-catalyzed transformation to the endo-thiol metabolite. If the latter occurs, it may favor H_2_S-release and contribute to the formation of downstream metabolites such as the M18H metabolite detected here.

A second unexpected finding based on current consensus of clopidogrel metabolism was the consistent detection of the parent molecule in urine samples across all four transplant cohorts, although we were aware of this based on (unpublished) pilot projects. In particular, the relative abundance observed in the cross-sectional KTR cohort was approximately ten times higher than in the longitudinal cohorts. Given that clopidogrel is known to undergo rapid hepatic metabolism under normal physiological conditions, its presence in urine at these levels is atypical [30,49,60]. Initially this finding was thought to be due to enzyme inhibitors, like the previously mentioned Kolliphor^®^ EL excipient in cyclosporine A (CsA) capsules as CsA is commonly prescribed in transplant recipients [69]. However, upon data analysis only one patient in the longitudinal cohort reported CsA use making this an unlikely cause of high parent drug abundance. Despite this, it should be kept in mind that the primary reference data stems from a mass balance study which included only 6 healthy volunteers and was conducted in the 1990s [30,31,60]. In that study, clopidogrel was not reported to be detected in urine, whereas all the clinical subgroups in the present investigation showed measurable amounts of the parent compound. In this regard, the detection of clopidogrel in this study may partly reflect advancements in analytical sensitivity achieved over the past three decades, although radioactivity-based detection techniques have traditionally been rather sensitive [70,71].

The growing sensitivity of analytical techniques may also have contributed to the detection of numerous low-abundance, uncharacterized metabolite signals. Among these, the *m*/*z* 356.07 signal stood out as particularly puzzling (Table 4), as it aligns with the masses of the thiol H1-4 metabolites [34]. Given their highly reactive nature and short half-lives, these thiol metabolites are not expected to appear in urine [72]. Alternatively, this *m*/*z* could correspond to an S-methyl derivative of CCA, analogous to the previously reported (inactive) S-methyl variant of prasugrel’s active thiol metabolite [73]. In addition to this, several other low-abundance features exhibited mass differences suggestive of glutathione-related conjugation patterns, such as cysteinylation. While glutathione’s role as a key reducing agent in clopidogrel bioactivation has been proposed, the appearance of such conjugation patterns in urine metabolites adds further evidence to the complexity of clopidogrel metabolism [43,48]. Together, these findings highlight how untargeted approaches can expand our understanding of the metabolic landscape of pharmaceuticals in real-world settings.

A strength of this study is that it applies untargeted pharmacometabolomics to real-world clopidogrel users across multiple transplant cohorts, thereby capturing (but not correcting for) a range of sources of variability. These sources include differences in drug use (e.g., dosage, therapy duration, adherence), as well as anthropometric, demographic, genetic, lifestyle, and physiological factors. We were furthermore able to confirm the presence of various expected metabolites, which may support existing pharmacokinetic modeling or pharmacogenetic testing approaches. In addition, we identified several previously unreported and uncharacterized metabolites, which could be considered in future efforts to refine these approaches. The newly described metabolites may also contribute to the ongoing effort to resolve analytical “dark matter” that still constitutes a large portion of features detected in untargeted metabolomics studies. Admittedly, most metabolite identifications achieved here correspond to level 3 confidence, or ‘putatively characterized compound classes’ according to the MSI [59]. However, all metabolites were only observed in clopidogrel-positive samples, adding evidence to their relevance as potential clopidogrel metabolites or, at least, clopidogrel-use-associated compounds.

Our study’s real-world design also introduces a key limitation, in that it inevitably captures variability we are unaware of and therefore cannot study in detail. Although this approach allows us to reflect real-world clopidogrel metabolism and excretion, it also introduces heterogeneity. While we observed considerable interindividual variation across transplant subgroups, the cohorts remain geographically homogenous, limiting the generalizability of our findings and emphasizing the need for replication in more diverse populations. Furthermore, we relied on biobanked samples and employed untargeted analytical techniques in a non-regulatory setting, both of which introduce potential (pre)analytical bias, including variability in sample handling, storage conditions, and timing of clopidogrel intake. These factors may influence metabolite presence and relative abundance. Lastly, the metabolite patterns detected may not fully reflect the real-time metabolite profile at the moment of urine collection, as they are influenced by various uncontrolled factors such as the dosing time during the 24 h collection period. Altogether, these limitations underscore the need to replicate our study in larger and better-characterized cohorts, ideally incorporating analytical workflows that allow for chiral separation and absolute quantification. Future research should also prioritize the targeted validation of these putative novel metabolites using fresh biospecimens and chemical reference standards. Expanding cohort diversity and integrating pharmacogenetic and clinical metadata will further clarify the underlying drivers of interindividual variability. Such insights will provide a more complete understanding of clopidogrel’s metabolism, which may support more personalized prescribing strategies and inform environmental assessments of pharmaceutical metabolites.

## 5. Conclusions

This study confirmed the presence of the established clopidogrel metabolites clopidogrel carboxylic acid (CCA) and its glucuronide (CAG) in urine, both as level 3 identifications (“putatively characterized compound classes”) according to the Metabolomics Standards Initiative (MSI). Besides various minor metabolite candidates, it furthermore reports the detection of four (MSI level 3) metabolites previously unreported in urine, namely the known M10 (previously found in human liver microsome experiments) and M18H (previously found in human plasma) metabolites, as well as a putatively ‘novel’ di-oxygenated version of CCA and mono-oxygenated version of CAG. Thereby, this study demonstrates the value of real-world drug metabolism studies in expanding our understanding of a drug’s pharmacokinetics. Our findings particularly illustrate how untargeted pharmacometabolomics can uncover metabolic transformations overlooked in mass balance studies, putatively offering a broader foundation for advancing pharmacogenetics and personalized medicine. Future research should, however, aim to include more diverse populations and integrate both genetic and nongenetic (e.g., comorbidities, lifestyle) risk factors to better define the determinants of metabolic variability. Targeted validation of novel metabolites using reference standards, chiral separation, and structural elucidation techniques may also be essential in this regard. Additionally, further studies could more thoroughly investigate the minor metabolites described in our work, which could indicate potential S-methylation of thiol metabolites and the involvement of glutathione in clopidogrel’s metabolism. Altogether, such efforts could enhance the clinical interpretability of pharmacometabolomic data, possibly contributing to more personalized prescribing and monitoring approaches of drug therapies in future healthcare practices.

## Figures and Tables

**Table 1:** Summary of sample characteristics of clopidogrel positive kidney and liver transplant recipients that are included in this study.

Characteristic As Median (IQR) or Percentage	Cross-Sectional Cohorts		Longitudinal Cohorts	
	KTR N = 12	LTR N = 7	KTR N = 26	LTR N = 9
Age (years)	57 (48–68)	62 (58–69)	66 (59–70)	60 (50–68)
Female sex	25	28	65	22
BMI (kg/m^2^)	25.6 (25.1–29.4)	27.7 (23.4–29.9)	27.8 (25.3–30.3)	30.5 (25.1–34.2)
eGFR (mL/min/1.73 m^2^)	51.2 (35.3–60.9)	78.8 (54.2–82.3)	54.7 (42.4–63.0)	72.5 (59.1–94.9)
Serum albumin (g/L)	43.5 (42.0–46.0)	45.0 (42.0–47.0)	45.5 (43.0–46.0)	40.0 (32.5–42.5)
Serum ALT (U/L)	19.0 (12.5–20.8)	24.0 (21.0–110.0)	18.0 (13.8–20.2)	24.0 (15.0–43.5)
Time since transplantation (months)	96.5 (42.8–183.8)	73.0 (60.0–144.0)	12.0 (9.8–24.0)	6.0 (3.0–9.0) *
*Self-reported medication use and smoker status*				
Clopidogrel use	100	100	92	89
Tacrolimus use	46	100	96	67
Mycophenolate mofetil use	33	14	96	33
Mycophenolic acid use	33	0	0	0
Azathioprine use	33	0	0	22
Statin use	83	71	62	56
ACE inhibitor use	33	14	19	11
Current smoker	36	43	80	67

Abbreviations: ACE, angiotensin-converting enzyme; ALT, alanine aminotransferase; BMI, body mass index; eGFR, estimated glomerular filtration rate; IQR, interquartile range; KTR, kidney transplant recipients; LTR, liver transplant recipients. Continuous and categorical data are presented as median (interquartile range, IQR) and valid percentage. * Four individuals were pre-transplant samples and excluded from this calculation.

**Table 2:** Overview of selected features based on automatic feature detection.

		Cross-Sectional Cohorts				Longitudinal Cohorts			
		KTR (N = 12)		LTR (N = 7)		KTR (N = 26)		LTR (N = 9)	
*m*/*z*	RT (min)	Rel. Median (%) ^a^	*p*-Value ^b^	Rel. Median (%) ^a^	*p*-Value ^b^	Rel. Median (%) ^a^	*p*-Value ^b^	Rel. Median (%) ^a^	*p*-Value ^b^
304.0188	7.5	37.5	5.3 × 10^−216^	19.0	1.1 × 10^−137^	25.7	1.8 × 10^−246^	27.2	9.8 × 10^−71^
306.0335	7.3	1.3	3.8 × 10^−154^	0.9 ^c^	6.7 × 10^−125^	0.9 ^c^	5.5 × 10^−64^	0.7 ^c^	1.6 × 10^−27^
308.0502	8.3	100.0	3.2 × 10^−219^	56.2	7.3 × 10^−69^	100.0	1.3 × 10^−197^	48.0	1.2 × 10^−42^
310.0830	5.7	3.8	4.2 × 10^−186^	2.3	2.0 × 10^−99^	2.5	1.3 × 10^−115^	4.0	1.2 × 10^−106^
322.0660	14.2	33.2	1.7 × 10^−192^	9.7	9.4 × 10^−86^	2.5	1.0 × 10^−112^	2.5	4.9 × 10^−55^
326.0602	6.2	0.9 ^c^	2.1 × 10^−125^	0.7 ^c^	2.3 × 10^−48^	0.8 ^c^	5.1 × 10^−98^	1.2	3.1 × 10^−52^
326.0605	7.9	5.2	3.8 × 10^−158^	2.3	3.7 × 10^−79^	3.2	1.5 × 10^−163^	4.2	3.4 × 10^−40^
328.0939	4.3	3.3	4.7 × 10^−111^	1.4	6.2 × 10^−50^	2.5	5.2 × 10^−130^	1.2	4.3 × 10^−29^
340.0396	7.1	2.8	2.8 × 10^−225^	1.8	6.0 × 10^−125^	2.3	1.8 × 10^−122^	1.7	3.2 × 10^−51^
340.0397	8.5	9.0	4.1 × 10^−202^	6.4	1.4 × 10^−129^	8.0	1.8 × 10^−194^	3.6	6.2 × 10^−58^
342.1100	8.8	4.1	5.8 × 10^−101^	2.4	2.0 × 10^−89^	5.6	1.1 × 10^−84^	7.6	1.0 × 10^−60^
342.1103	7.7	13.3	4.8 × 10^−144^	7.4	1.5 × 10^−175^	10.4	7.1 × 10^−90^	16.2	2.9 × 10^−60^
356.0696	7.4	2.1	2.5 × 10^−161^	1.2	8.4 × 10^−142^	1.7	2.9 × 10^−153^	1.4	3.8 × 10^−61^
356.0703	8.2	3.0	1.4 × 10^−194^	1.6	1.2 × 10^−147^	2.3	3.9 × 10^−165^	2.1	4.5 × 10^−90^
386.0807	10.8	0.0 ^b^	1.7 × 10^−44^	0.1 ^c^	3.4 × 10^−41^	0.2 ^c^	1.3 × 10^−32^	1.1	4.5 × 10^−46^
386.0811	10.0	not detected	-	not detected	-	not detected	-	5.4	7.8 × 10^−77^
386.0824	10.3	2.4	1.7 × 10^−128^	1.4	1.8 × 10^−104^	1.8	2.1 × 10^−69^	not detected	
427.0538	5.4	1.2	1.3 × 10^−109^	0.5 ^c^	1.1 × 10^−37^	1.0	1.6 × 10^−84^	3.4	5.2 × 10^−59^
445.0624	3.0	0.3 ^c^	3.9 × 10^−59^	0.2 ^c^	2.3 × 10^−50^	0.3 ^c^	1.7 × 10^−53^	3.9	2.4 × 10^−31^
459.0797	9.1	1.2	1.3 × 10^−55^	1.1	3.0 × 10^−78^	1.4	3.8 × 10^−30^	11.8	4.7 × 10^−25^
461.0583	4.9	1.9	1.3 × 10^−155^	0.8 ^b^	1.2 × 10^−51^	1.5	7.1 × 10^−103^	3.8	1.6 × 10^−52^
484.0806	11.5	88.5	2.5 × 10^−148^	100.0	3.7 × 10^−101^	82.5	1.5 × 10^−133^	100.0	1.8 × 10^−40^
484.0812	10.8	7.5	1.7 × 10^−34^	10.6	2.5 × 10^−38^	7.1	9.0 × 10^−113^	21.1	2.4 × 10^−23^
500.0758	10.2	23.3	8.5 × 10^−127^	4.8	1.5 × 10^−26^	23.3	2.9 × 10^−153^	29.6	2.3 × 10^−52^
502.1082	8.2	6.7	9.1 × 10^−198^	4.4	1.9 × 10^−187^	5.1	9.3 × 10^−180^	3.0	2.5 × 10^−73^
514.0913	9.9	0.3 ^c^	2.3 × 10^−56^	0.3 ^c^	8.9 × 10^−53^	0.0 ^c^	4.0 × 10^−24^	2.5	2.9 × 10^−43^
514.0919	11.5	3.8	2.3 × 10^−132^	2.9	3.6 × 10^−49^	2.4	7.8 × 10^−110^	7.6	2.3 × 10^−34^
516.0703	6.6	2.4	7.4 × 10^−107^	2.7	2.7 × 10^−111^	1.9	7.7 × 10^−159^	2.6	6.9 × 10^−50^
516.0713	9.4	0.1 ^c^	7.4 × 10^−36^	5.0	9.4 × 10^−72^	0.0 ^c^	7.0 × 10^−12^	3.2	1.8 × 10^−46^
516.1072	8.6	0.5 ^c^	3.4 × 10^−77^	0.8 ^c^	2.2 × 10^−49^	0.8 ^c^	1.1 × 10^−41^	2.1	1.7 × 10^−28^
518.1389	7.9	1.1	8.0 × 10^−140^	0.7 ^c^	3.1 × 10^−192^	0.8 ^c^	1.8 × 10^−67^	0.8 ^c^	9.8 × 10^−55^
530.0848	8.2	0.4 ^c^	1.8 × 10^−45^	4.2	1.3 × 10^−53^	1.1	3.7 × 10^−55^	1.1	6.6 × 10^−20^
546.1180	12.0	0.9 ^c^	6.9 × 10^−46^	1.2	8.2 × 10^−53^	1.2	4.3 × 10^−79^	3.4	4.8 × 10^−49^
546.1189	10.5	2.7	2.4 × 10^−44^	2.7	1.9 × 10^−53^	4.9	5.8 × 10^−44^	14.7	3.6 × 10^−32^
637.0896	7.3	8.0	2.2 × 10^−124^	4.1	2.7 × 10^−129^	4.0	4.5 × 10^−142^	9.5	6.7 × 10^−65^
647.0799	9.3	0.9 ^c^	1.6 × 10^−56^	1.7	1.3 × 10^−86^	0.0 ^c^	3.2 × 10^−40^	1.0	8.2 × 10^−33^
647.0815	10.3	1.4	1.5 × 10^−86^	1.0	1.3 × 10^−128^	0.6 ^c^	1.4 × 10^−124^	1.6	4.5 × 10^−36^
823.1127	9.2	1.5	7.6 × 10^−97^	2.7	1.0 × 10^−80^	0.8 ^c^	2.1 × 10^−116^	1.8	5.3 × 10^−41^

Abbreviations: KTR, kidney transplant recipients; LTR, liver transplant recipients; *m*/*z*, mass-to-charge ratio; RT, retention time; rel., relative. ^a^ For each cohort, the highest observed median intensity value (in the clopidogrel users’ group) was set at 100%, and all other median values were expressed relative to this highest value. ^b^ *p* values are from the statistical comparison between exposure-positive and exposure-negative samples using a *t*-test (α = 0.05, with application of Bonferroni correction). ^c^ These features were below the abundance threshold for the respective cohort but are shown because they were included in the other cohort.

**Table 3:** Characteristics of the selected features, based on manual assessment of and data extraction from the raw pharmacometabolomics data.

		All 4 Cohorts	Cross-Sectional Cohorts		Longitudinal Cohorts	
			KTR (N = 13)	LTR (N = 7)	KTR (N = 26)	LTR (N = 9)
*m*/*z*	RT (min)	Rel. Median (%) ^a^	Rel. Median (%) ^b^	Rel. Median (%) ^b^	Rel. Median (%) ^b^	Rel. Median (%) ^b^
304.02	7.5	27.8	35.2	19.3	25.5	22.6
306.03	7.5	2.4	2.8	2.4	2.0	1.6
308.05	8.3	100.0	96.1	59.2	100.0	38.2
310.08	5.7	3.1	3.2	2.0	2.4	2.9
322.07	14.2	5.8	32.2	10.5	2.5	2.2
326.06	6.2, 7.9	3.8	5.1	2.3	3.3	3.6
328.09	4.2	1.7	1.8	0.7	1.7	0.8
340.04	7.1, 8.5	8.9	8.1	7.3	8.4	3.3
342.11	7.6, 8.8	14.3	14.6	8.7	12.7	18.0
356.07	7.4, 8.2	3.8	3.7	2.1	3.4	2.6
386.08	10.0, 10.3, 10.6, 10.8	3.3	3.8	1.6	2.5	4.5
427.05	5.4	0.8	0.8	0.4	0.7	1.7
445.06	3.0, 3.2, 3.5, 3.8	0.8	0.8	0.6	0.6	2.4
459.08	8.6, 9.1, 9.6	0.9	1.0	0.8	0.8	6.3
461.06	4.9	1.5	1.4	1.0	1.2	1.8
484.08	10.6, 11.0, 11.3, 11.6	83.8	100.0	100.0	77.5	100.0
500.08	10.2	14.2	13.0	2.8	15.2	16.6
502.11	8.2	4.4	4.9	3.2	3.8	2.0
514.09	9.9, 11.2, 11.5	2.7	2.8	1.9	2.2	4.9
516.07	6.6, 9.4	2.7	2.9	3.3	1.9	2.7
516.11	8.5, 8.7, 8.9	0.8	0.9	0.7	0.8	1.2
518.14	7.9	0.7	0.7	0.4	0.5	0.5
530.08	8.2	1.6	1.1	2.3	1.4	1.0
546.12	10.5, 11.4, 12.0	3.8	2.5	2.2	3.4	7.2
637.09	7.3	4.3	5.9	3.1	3.5	4.7
647.08 ^c^	6.7, 6.9	0.1	0.1	0.1	0.1	0.0
823.11	8.6, 9.0, 9.3, 10.3	0.8	1.5	1.8	0.6	2.0

Abbreviations: KTR, kidney transplant recipients; LTR, liver transplant recipients; *m*/*z*, mass-to-charge ratio; RT, retention time; rel., relative. ^a^ For data of all cohorts combined, the highest observed median intensity value (in the clopidogrel users’ group) was set at 100%, and all other median values were expressed relative to this highest value. ^b^ For each cohort, the highest observed median intensity value (in the clopidogrel users’ group) was set at 100%, and all other median values were expressed relative to this highest value. ^c^ Feature dropped for further analysis due to very low abundance.

**Table 4:** Relative metabolite abundance data of clopidogrel and an overview of its putative metabolites (sorted from large to small based on the data from all cohorts combined).

				Median Metabolite Abundance (%) ^a^					
				Cross-Sectional Cohorts			Longitudinal Cohorts		
Possible Identity ^b,c^	Possible Molecular Formula	*m*/*z*	RT (min)	All (N = 55)	KTR (N = 13)	LTR (N = 7)		KTR (N = 26)	LTR (N = 9)
Clopidogrel carboxylic acid (CCA)	C_15_H_14_ClNO_2_S	308.05	8.3	29.6	26.6	19.3		33.6	25.1
Clopidogrel carboxylic acid glucuronide (CAG)	C_21_H_22_ClNO_8_S	484.08	10.6, 11.0, 11.3, 11.6	29.0	29.6	38.5		27.2	33.6
Doubly oxidized CCA (M10)	C_15_H_11_ClNO_2_S	304.02	7.5	8.6	9.3	8.2		9.0	5.1
Clopidogrel-thiol-derived secondary alcohol (M18H)	C_16_H_20_ClNO_5_	342.11	7.6, 8.8	4.3	3.5	3.4		4.9	4.5
Mono-oxygenated CAG	C_21_H_22_ClNO_9_S	500.08	10.2	4.3	3.9	2.6		4.5	3.4
Di-oxygenated CCA	C_15_H_14_ClNO_4_S	340.04	7.1, 8.5	2.3	2.6	2.7		2.8	1.18
Clopidogrel	C_16_H_16_ClNO_2_S	322.07	14.2	1.34	10.3	4.0		0.90	0.58
Minor metabolite 502.11		502.11	8.2	1.25	1.5	1.27		1.23	0.92
Minor metabolite 326.06		326.06	6.2, 7.9	1.18	1.18	1.25		1.18	0.92
Minor metabolite 637.09		637.09	7.3	1.10	1.40	1.36		0.99	1.7
Minor metabolite 356.07		356.07	7.4, 8.2	1.06	1.37	0.82		1.20	0.68
Minor metabolite 546.12		546.12	10.5, 11.4, 12.0	1.03	0.73	0.98		0.76	3.5
Minor metabolite 386.08		386.08	10.0, 10.3, 10.6, 10.8	0.96	1.06	0.64		0.91	1.46
Minor metabolite 310.08		310.08	5.7	0.87	0.98	0.80		0.86	1.18
Minor metabolite 514.09		514.09	9.9, 11.2, 11.5	0.75	0.71	0.50		0.73	1.8
Minor metabolite 516.07		516.07	6.6, 9.4	0.75	0.97	1.46		0.61	0.73
Minor metabolite 306.03		306.03	7.3	0.71	0.79	0.79		0.61	0.54
Minor metabolite 328.09		328.09	4.2	0.47	0.44	0.26		0.49	0.36
Minor metabolite 530.08		530.08	8.2	0.45	0.40	0.52		0.46	0.71
Minor metabolite 461.06		461.06	4.9	0.41	0.39	0.38		0.39	0.74
Minor metabolite 459.08		459.08	8.6, 9.1, 9.6	0.27	0.24	0.27		0.27	2.4
Minor metabolite 427.05		427.05	5.4	0.26	0.30	0.18		0.23	0.74
Minor metabolite 516.11		516.11	8.5, 8.7, 8.9	0.24	0.21	0.24		0.26	0.54
Minor metabolite 823.11		823.11	8.6, 9.0, 9.3, 10.3	0.24	0.41	0.70		0.20	0.44
Minor metabolite 445.06		445.06	3.0, 3.2, 3.5, 3.8	0.22	0.27	0.18		0.15	0.61
Minor metabolite 518.14		518.14	7.9	0.18	0.18	0.16		0.18	0.18

Abbreviations: KTR, kidney transplant recipients; LTR, liver transplant recipients; *m*/*z*, mass-to-charge ratio; RT, retention time; rel., relative. ^a^ Median metabolite abundance values presented in the table reflect the median values for all studies combined (first column) or per cohort (second to fifth column) of the relative quantitative readouts that were calculated by dividing the signal intensity of each substance individually by the sum of signal intensities of all substances found per clopidogrel user. ^b^ Solely clopidogrel reflects a substance for which the identity was verified by spectral library matching. All other identities reflect putatively identified metabolites and are based upon spectral similarity with known substances. ^c^ Exemplary extracted ion chromatograms and fragment spectra are shown in the Supplementary Figures S3–S28, and information on mass accuracy of the 7 prioritized substances as well as on various candidate fragment ions detected across these substances is shown in the Supplementary Tables S3–S9.

## Data Availability

All the pharmacometabolomic data have been deposited in an open-access data repository, which can be found at https://doi.org/10.26037/yareta:64ruex2sxff5nenyfyexurzs3m (accessed on 19 February 2026).

## Supplementary Materials

The following supporting information can be downloaded at: https://www.mdpi.com/article/10.3390/metabo16030210/s1. Method S1: PubMed search strategy. Figure S1: PRISMA flowchart of study inclusion. Figure S2: Exemplary spectral library matching-based identification of clopidogrel (CID 60606) which was observed in the urine of a kidney transplant recipient who declared usage of this drug. Figure S3: (**A**) MS1-level extracted ion chromatogram and (**B**) SWATH/MS fragment spectrum of clopidogrel observed in urine of a human clopidogrel user. Figure S4: (**A**) MS1-level extracted ion chromatogram and (**B**) SWATH/MS fragment spectrum of a putative clopidogrel metabolite with an *m*/*z* value of 304.02 observed in urine of a human clopidogrel user. Figure S5: (**A**) MS1-level extracted ion chromatogram and (**B**) SWATH/MS fragment spectrum of a putative clopidogrel metabolite with an *m*/*z* value of 306.03 observed in urine of a human clopidogrel user. Figure S6: (**A**) MS1-level extracted ion chromatogram and (**B**) SWATH/MS fragment spectrum of a putative clopidogrel metabolite with an *m*/*z* value of 308.05 observed in urine of a human clopidogrel user. Figure S7: (**A**) MS1-level extracted ion chromatogram and (**B**) SWATH/MS fragment spectrum of a putative clopidogrel metabolite with an *m*/*z* value of 310.08 observed in urine of a human clopidogrel user. Figure S8: (**A**) MS1-level extracted ion chromatogram and (**B**,**C**) SWATH/MS fragment spectra of putative clopidogrel metabolites with an *m*/*z* value of 326.06 observed in urine of a human clopidogrel user. Figure S9: (**A**) MS1-level extracted ion chromatogram and (**B**) SWATH/MS fragment spectrum of a putative clopidogrel metabolite with an *m*/*z* value of 328.09 observed in urine of a human clopidogrel user. Figure S10: (**A**) MS1-level extracted ion chromatogram and (**B**,**C**) SWATH/MS fragment spectra of putative clopidogrel metabolites with an *m*/*z* value of 340.04 observed in urine of a human clopidogrel user. Figure S11: (**A**) MS1-level extracted ion chromatogram and (**B**,**C**) SWATH/MS fragment spectra of putative clopidogrel metabolites with an *m*/*z* value of 342.11 observed in urine of a human clopidogrel user. Figure S12: (**A**) MS1-level extracted ion chromatogram and (**B**,**C**) SWATH/MS fragment spectra of putative clopidogrel metabolites with an *m*/*z* value of 356.07 observed in urine of a human clopidogrel user. Figure S13: (**A**) MS1-level extracted ion chromatogram and (**B**–**E**) SWATH/MS fragment spectra of putative clopidogrel metabolites with an *m*/*z* value of 386.08 observed in urine of a human clopidogrel user. Figure S14: (**A**) MS1-level extracted ion chromatogram and (**B**) SWATH/MS fragment spectrum of a putative clopidogrel metabolite with an *m*/*z* value of 427.05 observed in urine of a human clopidogrel user. Figure S15: (**A**) MS1-level extracted ion chromatogram and (**B**–**E**) SWATH/MS fragment spectra of putative clopidogrel metabolites with an *m*/*z* value of 445.06 observed in urine of a human clopidogrel user. Figure S16: (**A**) MS1-level extracted ion chromatogram and (**B**–**D**) SWATH/MS fragment spectra of putative clopidogrel metabolites with an *m*/*z* value of 459.08 observed in urine of a human clopidogrel user. Figure S17: (**A**) MS1-level extracted ion chromatogram and (**B**) SWATH/MS fragment spectrum of a putative clopidogrel metabolite with an *m*/*z* value of 461.06 observed in urine of a human clopidogrel user. Figure S18: (**A**) MS1-level extracted ion chromatogram and (**B**–**E**) SWATH/MS fragment spectra of putative clopidogrel metabolites with an *m*/*z* value of 484.08 observed in urine of a human clopidogrel user. Figure S19: (**A**) MS1-level extracted ion chromatogram and (**B**) SWATH/MS fragment spectrum of a putative clopidogrel metabolite with an *m*/*z* value of 500.08 observed in urine of a human clopidogrel user. Figure S20: (**A**) MS1-level extracted ion chromatogram and (**B**) SWATH/MS fragment spectrum of a putative clopidogrel metabolite with an *m*/*z* value of 502.11 observed in urine of a human clopidogrel user. Figure S21: (**A**) MS1-level extracted ion chromatogram and (**B**–**D**) SWATH/MS fragment spectra of putative clopidogrel metabolites with an *m*/*z* value of 514.09 observed in urine of a human clopidogrel user. Figure S22: (**A**) MS1-level extracted ion chromatogram and (**B**,**C**) SWATH/MS fragment spectra of putative clopidogrel metabolites with an *m*/*z* value of 516.07 observed in urine of a human clopidogrel user. Figure S23: (**A**) MS1-level extracted ion chromatogram and (**B**–D) SWATH/MS fragment spectra of putative clopidogrel metabolites with an *m*/*z* value of 516.11 observed in urine of a human clopidogrel user. Figure S24: (**A**) MS1-level extracted ion chromatogram and (**B**) SWATH/MS fragment spectrum of a putative clopidogrel metabolite with an *m*/*z* value of 518.14 observed in urine of a human clopidogrel user. Figure S25: (**A**) MS1-level extracted ion chromatogram and (**B**) SWATH/MS fragment spectrum of a putative clopidogrel metabolite with an *m*/*z* value of 530.08 observed in urine of a human clopidogrel user. Figure S26: (**A**) MS1-level extracted ion chromatogram and (**B**–**D**) SWATH/MS fragment spectra of putative clopidogrel metabolites with an *m*/*z* value of 546.12 observed in urine of a human clopidogrel user. Figure S27: (**A**) MS1-level extracted ion chromatogram and (**B**) SWATH/MS fragment spectrum of a putative clopidogrel metabolite with an *m*/*z* value of 637.09 observed in urine of a human clopidogrel user. Figure S28: (**A**) MS1-level extracted ion chromatogram and (**B**–**E**) SWATH/MS fragment spectra of putative clopidogrel metabolites with an *m*/*z* value of 823.11 observed in urine of a human clopidogrel user. Table S1: Overview of MarkerView data (pre)processing settings. Table S2: Overview of manually integrated feature signals. Table S3: Overview of representative (candidate) fragments of clopidogrel (MSI level 2 identification), as depicted in Figure S3. Table S4: Overview of representative (candidate) fragments of the putative doubly oxidized clopidogrel carboxylic acid metabolite (M10; MSI level 3 identification), as depicted in Figure S4. Table S5: Overview of representative (candidate) fragments of the putative clopidogrel carboxylic acid metabolite (MSI level 3 identification), as depicted in Figure S6. Table S6: Overview of representative (candidate) fragments of two putative di-oxygenated clopidogrel carboxylic acid metabolites (MSI level 3 identification), as depicted in Figure S10. Table S7: Overview of representative (candidate) fragments of two putative clopidogrel-thiol-derived secondary alcohol metabolites (M18H; MSI level 3 identification), as depicted in Figure S11. Table S8: Overview of representative (candidate) fragments of four putative clopidogrel carboxylic acid glucuronide metabolites (MSI level 3 identification), as depicted in Figure S18. Table S9: Overview of representative (candidate) fragments of the putative mono-oxygenated clopidogrel carboxylic acid glucuronide metabolite (MSI level 3 identification), as depicted in Figure S19.

## Abbreviations

The following abbreviations are used in this manuscript:

ACE	Angiotensin-Converting Enzyme
ALT	Alanine Transaminase
BMI	Body Mass Index
CAG	Clopidogrel Carboxylic Acid Glucuronide
CCA	Clopidogrel Carboxylic Acid
CES1	Carboxylesterase 1
CKD-EPI	Chronic Kidney Disease Epidemiology Collaboration
CPIC	Clinical Pharmacogenetics Implementation Consortium
CsA	Cyclosporine A
CYP	Cytochrome P450
DIA	Data-Independent Acquisition
eGFR	estimated Glomerular Filtration Rate
HR	High Resolution
KTR	Kidney Transplant Recipient
LC	Liquid Chromatography
LTR	Liver Transplant Recipient
MS	Mass Spectrometry
MSI	Metabolomic Standards Initiative
*m*/*z*	Mass-to-charge
PCA	Principal Component Analysis
P2Y12	P2Y Purinoreceptor 12
PGx	Pharmacogenetics
PON1	Paraoxonase-1
PMx	Pharmacometabolomics
RT	Retention Time
