# Supplementary material for: Pharmacometabolomics Detects Unreported Clopidogrel Metabolites in the Urine of Kidney and Liver Transplant Recipients
Source: Metabolites. 2026 Mar 21;16(3):210. doi: 10.3390/metabo16030210 (PMC13028800; doi:10.3390/metabo16030210)
Supplement: Supplementary file 1 [file metabolites-16-00210-s001.zip › metabolites-4189979-supplementary.pdf]

Pharmacometabolomics detects unreported clopidogrel metabolites in the urine of kidney and liver transplant recipients

Cassandra Piccolotto[1], Stephan J. L. Bakker[2], Vincent E. de Meijer[3], Gérard Hopfgartner[4], Peter Fodran[5], Frank Klont[1,6,*], and TransplantLines Investigators[7,†]

[1]Unit of PharmacoTherapy, -Epidemiology & -Economics, Groningen Research Institute of Pharmacy, University of Groningen, Antonius Deusinglaan 1, 9713 AV Groningen, The Netherlands
[2]Division of Nephrology, Department of Internal Medicine, University Medical Center Groningen, University of Groningen, Hanzeplein 1, 9713 GZ Groningen, The Netherlands
[3]Division of Hepatobiliary Surgery and Liver Transplantation, Department of Surgery, University Medical Center Groningen, University of Groningen, Hanzeplein 1, 9713 GZ Groningen, The Netherlands
[4]Life Sciences Mass Spectrometry, Department of Inorganic and Analytical Chemistry, University of Geneva, Quai Ernest Ansermet 30, 1211 Genève, Switzerland
[5]Department of Chemical and Pharmaceutical Biology, Groningen Research Institute of Pharmacy, University of Groningen, Antonius Deusinglaan 1, 9713 AV Groningen, The Netherlands
[6]Department of Clinical Pharmacy and Pharmacology, University Medical Center Groningen, University of Groningen, Hanzeplein 1, 9713 GZ Groningen, The Netherlands
[7]Group of Authors on Behalf of the Transplant Lines Biobank and Cohort Study, University Medical Center Groningen, University of Groningen, Hanzeplein 1, 9713 GZ Groningen, The Netherlands
*Author to whom correspondence should be addressed.
†Collaborators/Membership of the TransplantLines Investigators is provided in the Acknowledgments.

Method S1. PubMed search strategy.

A literature search was conducted on PubMed and Embase using the search terms “clopidogrel”, “R-130964”, “R130964”, ”SC-25989C”, “SC25989C”, “SC 25989C”, “SC-25990C”, “SC25990C”, “SC 25990C”, “SR-25989”, “SR25989”, “SR 25989”, “PCR-4099”, “PCR4099”, “PCR 4099” AND “metabol*”, “biotransform*” AND “mass balance”, “excretion balance”, “radioact*”, “ADME”, “microsome*”, "S9 fraction", "liver S9", “hepatocyte*” in the title and/or abstract. The “*” indicates which terms used the truncated form and no time restrictions or filters were applied. The last search was performed on April 3rd, 2025.

Table S1. Overview of MarkerView data (pre)processing settings.

Setting	Value
Feature finding:	
Experiment	MS1
Minimum retention time	0.50 min
Maximum retention time	16.00 min
Subtraction offset	15 scans*
Subtraction multiplication factor	1.3*
Noise threshold	5*
Minimum spectral peak width	5 ppm*
Minimum retention time peak width	5 scans*
Assign charge states	Enabled*
Feature alignment:	
Retention time tolerance	0.50 min
Mass tolerance	0.01 Da
Feature filtering:	
Maximum number of peaks	8,000,000**
Remove peaks in < N samples	Disabled***
Isotope filtering	Disabled****
Intensity threshold	5
Use exclusion list	Disabled*****
Retention time filtering	Disabled***
Use area integrated from raw data, not from original peak finding	Disabled*
Principle component analysis:	
PCA preprocessing - Weighting	None*
PCA preprocessing - Scaling	Pareto*
Perform PCA-DA (supervised)	Disabled
T-test:	
Samples per group for “first to last” comparison	Disabled*
Use Welch t-test	Disabled*

*: default setting, proposed by the software developer.
**: This parameter was set high enough to prevent peaks from getting filtered at this stage.
***: The corresponding filtering step is not needed due to the primary filtering based differential analysis between exposure-positive and exposure-negative samples.
****: This filtering step was omitted, because isotope filtering is also done manually at a later stage (during which it actually can support the presumption that a prioritized monoisotopic feature is a clopidogrel metabolite, notably by being accompanied by the expected isotopic signals).
*****: The corresponding filtering step could, for example, be used to remove internal standard or blank signals, yet this is not needed due to the primary filtering based differential analysis between exposure- positive and exposure-negative samples.

Table S2. Overview of manually integrated feature signals.

Feature *m/z* (see Table 3)	Signal trace for peak area integration	*m/z* range
322.07	MS1-level quantifier trace (precursor)	322.0613 - 322.0713
	MS2-level qualifier trace (residual precursor)	322.0613 - 322.0713
	MS2-level qualifier trace (fragment ion)	155.0208 - 155.0308
304.02	MS1-level quantifier trace (precursor)	304.0138 - 304.0238
	MS2-level qualifier trace (residual precursor)	304.0138 - 304.0238
	MS2-level qualifier trace (fragment ion)	136.0165 - 136.0265
306.03	MS1-level quantifier trace (precursor)	306.0285 - 306.0385
	MS2-level qualifier trace (residual precursor)	306.0285 - 306.0385
	MS2-level qualifier trace (fragment ion)	169.0001 - 169.0101
308.05	MS1-level quantifier trace (precursor)	308.0452 - 308.0552
	MS2-level qualifier trace (residual precursor)	308.0452 - 308.0552
	MS2-level qualifier trace (fragment ion)	125.0103 - 125.0203
310.08	MS1-level quantifier trace (precursor)	310.0780 - 310.0880
	MS2-level qualifier trace (residual precursor)	310.0780 - 310.0880
	MS2-level qualifier trace (fragment ion)	125.0103 - 125.0203
326.06	MS1-level quantifier trace (precursor)	326.0555 - 326.0655
	MS2-level qualifier trace (residual precursor)	326.0555 - 326.0655
	MS2-level qualifier trace (fragment ion), 6.2 min.	198.0266 - 198.0366
	MS2-level qualifier trace (fragment ion), 7.9 min.	198.0266 - 198.0366
328.09	MS1-level quantifier trace (precursor)	328.0889 - 328.0989
	MS2-level qualifier trace (residual precursor)	328.0889 - 328.0989
	MS2-level qualifier trace (fragment ion)	169.0001 - 169.0101
340.04	MS1-level quantifier trace (precursor)	340.0347 - 340.0447
	MS2-level qualifier trace (residual precursor)	340.0347 - 340.0447
	MS2-level qualifier trace (fragment ion), 7.1 min.	113.0103 - 113.0203
	MS2-level qualifier trace (fragment ion), 8.5 min.	125.0103 - 125.0203
342.11	MS1-level quantifier trace (precursor)	342.1050 - 342.1150
	MS2-level qualifier trace (residual precursor)	342.1050 - 342.1150
	MS2-level qualifier trace (fragment ion), 7.6 min.	155.0208 - 155.0308
	MS2-level qualifier trace (fragment ion), 8.8 min.	155.0208 - 155.0308
356.07	MS1-level quantifier trace (precursor)	356.0646 - 356.0746
	MS2-level qualifier trace (residual precursor)	356.0646 - 356.0746
	MS2-level qualifier trace (fragment ion), 7.4 min.	125.0103 - 125.0203
	MS2-level qualifier trace (fragment ion), 8.2 min.	125.0103 - 125.0203
386.08	MS1-level quantifier trace (precursor)	386.0761 - 386.0861
	MS2-level qualifier trace (residual precursor)	386.0761 - 386.0861
	MS2-level qualifier trace (fragment ion), 10.0 min.	155.0208 - 155.0308
	MS2-level qualifier trace (fragment ion), 10.3 min.	155.0208 - 155.0308
	MS2-level qualifier trace (fragment ion), 10.6 min.	155.0208 - 155.0308
	MS2-level qualifier trace (fragment ion), 10.8 min.	155.0208 - 155.0308
427.05	MS1-level quantifier trace (precursor)	427.0488 - 427.0588
	MS2-level qualifier trace (residual precursor)	427.0488 - 427.0588
	MS2-level qualifier trace (fragment ion)	125.0103 - 125.0203
445.06	MS1-level quantifier trace (precursor)	445.0574 - 445.0674
	MS2-level qualifier trace (residual precursor)	445.0574 - 445.0674
	MS2-level qualifier trace (fragment ion), 3.0 min.	125.0103 - 125.0203
	MS2-level qualifier trace (fragment ion), 3.2 min.	125.0103 - 125.0203
	MS2-level qualifier trace (fragment ion), 3.5 min.	125.0103 - 125.0203
	MS2-level qualifier trace (fragment ion), 3.8 min.	125.0103 - 125.0203
459.08	MS1-level quantifier trace (precursor)	459.0747 - 459.0847
	MS2-level qualifier trace (residual precursor)	459.0747 - 459.0847
	MS2-level qualifier trace (fragment ion), 8.6 min.	155.0208 - 155.0308
	MS2-level qualifier trace (fragment ion), 9.1 min.	155.0208 - 155.0308
	MS2-level qualifier trace (fragment ion), 9.6 min.	155.0208 - 155.0308
461.06	MS1-level quantifier trace (precursor)	461.0533 - 461.0633
	MS2-level qualifier trace (residual precursor)	461.0533 - 461.0633
	MS2-level qualifier trace (fragment ion)	169.0001 - 169.0101
484.08	MS1-level quantifier trace (precursor)	484.0756 - 484.0856
	MS2-level qualifier trace (residual precursor)	484.0756 - 484.0856
	MS2-level qualifier trace (fragment ion), 10.6 min.	152.0212 - 152.0312
	MS2-level qualifier trace (fragment ion), 11.0 min.	152.0212 - 152.0312
	MS2-level qualifier trace (fragment ion), 11.3 min.	152.0212 - 152.0312
	MS2-level qualifier trace (fragment ion), 11.6 min.	152.0212 - 152.0312
500.08	MS1-level quantifier trace (precursor)	500.0708 - 500.0808
	MS2-level qualifier trace (residual precursor)	500.0708 - 500.0808
	MS2-level qualifier trace (fragment ion)	152.0212 - 152.0312
502.11	MS1-level quantifier trace (precursor)	502.1032 - 502.1132

	MS2-level qualifier trace (residual precursor)	502.1032 - 502.1132
	MS2-level qualifier trace (fragment ion)	198.0266 - 198.0366
514.09	MS1-level quantifier trace (precursor)	514.0869 - 514.0969
	MS2-level qualifier trace (residual precursor)	514.0869 - 514.0969
	MS2-level qualifier trace (fragment ion), 9.9 min.	125.0103 - 125.0203
	MS2-level qualifier trace (fragment ion), 11.2 min.	125.0103 - 125.0203
	MS2-level qualifier trace (fragment ion), 11.5 min.	125.0103 - 125.0203
516.07	MS1-level quantifier trace (precursor)	516.0663 - 516.0763
	MS2-level qualifier trace (residual precursor)	516.0663 - 516.0763
	MS2-level qualifier trace (fragment ion), 6.6 min.	169.0001 - 169.0101
	MS2-level qualifier trace (fragment ion), 9.4 min.	169.0001 - 169.0101
516.11	MS1-level quantifier trace (precursor)	516.1022 - 516.1122
	MS2-level qualifier trace (residual precursor)	516.1022 - 516.1122
	MS2-level qualifier trace (fragment ion), 8.5 min.	155.0208 - 155.0308
	MS2-level qualifier trace (fragment ion), 8.7 min.	155.0208 - 155.0308
	MS2-level qualifier trace (fragment ion), 8.9 min.	155.0208 - 155.0308
518.14	MS1-level quantifier trace (precursor)	518.1339 - 518.1439
	MS2-level qualifier trace (residual precursor)	518.1339 - 518.1439
	MS2-level qualifier trace (fragment ion)	155.0208 - 155.0308
530.08	MS1-level quantifier trace (precursor)	530.0798 - 530.0898
	MS2-level qualifier trace (residual precursor)	530.0798 - 530.0898
	MS2-level qualifier trace (fragment ion)	155.0208 - 155.0308
546.12	MS1-level quantifier trace (precursor)	546.1139 - 546.1239
	MS2-level qualifier trace (residual precursor)	546.1139 - 546.1239
	MS2-level qualifier trace (fragment ion), 10.5 min.	155.0208 - 155.0308
	MS2-level qualifier trace (fragment ion), 11.4 min.	155.0208 - 155.0308
	MS2-level qualifier trace (fragment ion), 12.0 min.	155.0208 - 155.0308
637.09	MS1-level quantifier trace (precursor)	637.0846 - 637.0946
	MS2-level qualifier trace (residual precursor)	637.0846 - 637.0946
	MS2-level qualifier trace (fragment ion)	169.0001 - 169.0101
823.11	MS1-level quantifier trace (precursor)	823.1077 - 823.1177
	MS2-level qualifier trace (residual precursor)	823.1077 - 823.1177
	MS2-level qualifier trace (fragment ion), 8.6 min.	125.0103 - 125.0203
	MS2-level qualifier trace (fragment ion), 9.0 min.	125.0103 - 125.0203
	MS2-level qualifier trace (fragment ion), 9.3 min.	125.0103 - 125.0203
	MS2-level qualifier trace (fragment ion), 10.3 min.	125.0103 - 125.0203

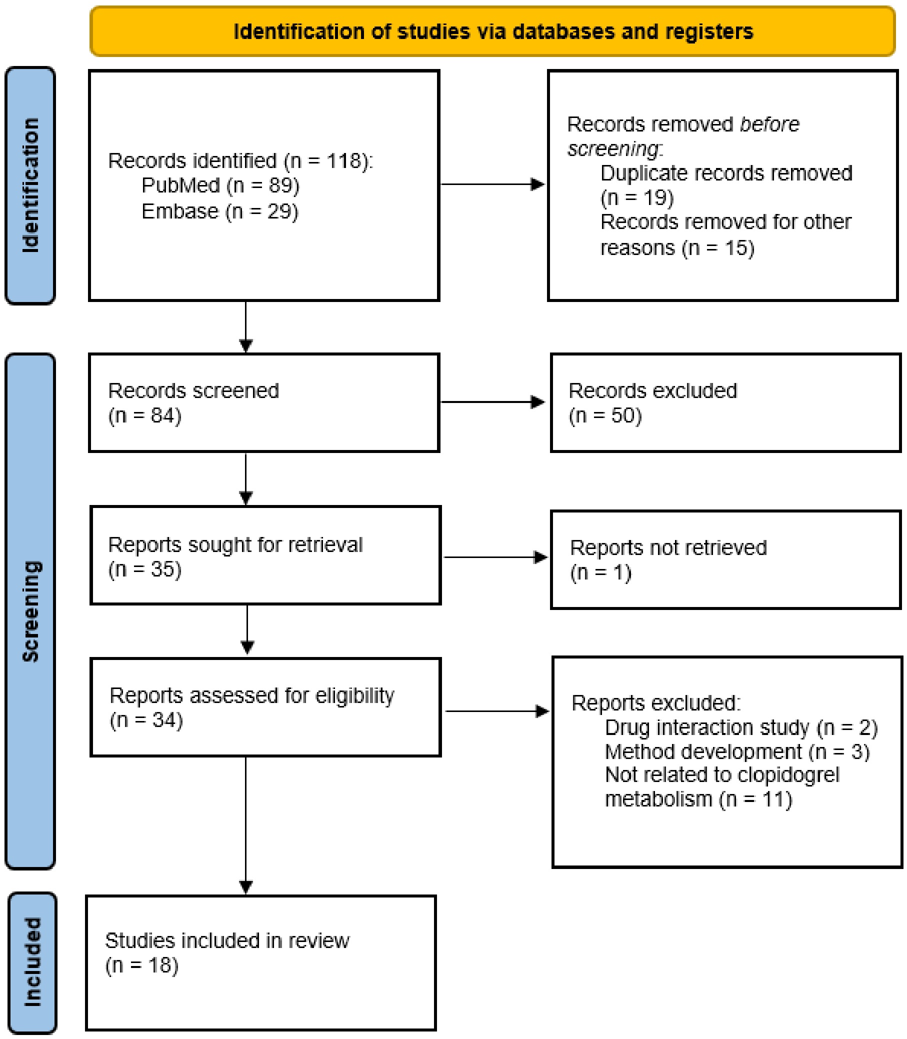

Figure S1. PRISMA flowchart of study inclusion. The 15 Articles removed for other reasons included reviews (5), abstracts (9), and case reports (1).

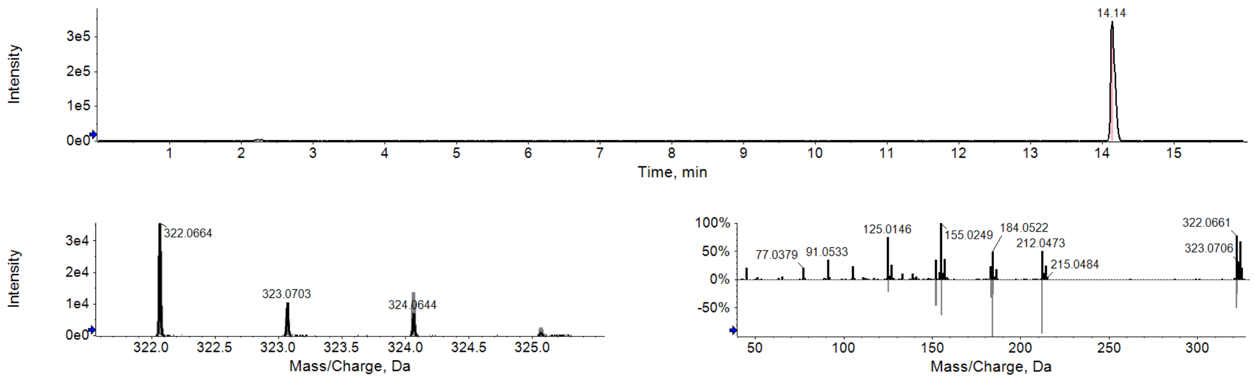

Figure S2. Exemplary spectral library matching-based identification of clopidogrel (CID 60606) which was observed in the urine of a kidney transplant recipient who declared usage of this drug. Regarding the data obtained and presented, these concern level 2 metabolite identifications according to the Metabolomics Standards Initiative (Sumner LW, Amberg A, Barrett D, *et al*. Proposed minimum reporting standards for chemical analysis Chemical Analysis Working Group (CAWG) Metabolomics Standards Initiative (MSI). Metabolomics 2007; 3: 211–221) for which we employed a commercial spectral library (SCIEX 'Forensic', version 1.1) and a commercial software tool (SCIEX PeakView, version 2.2.0.11391).

A. MS1-level extracted ion chromatogram (m/z 322.0663 ± 0.0125) of an exemplary clopidogrel user

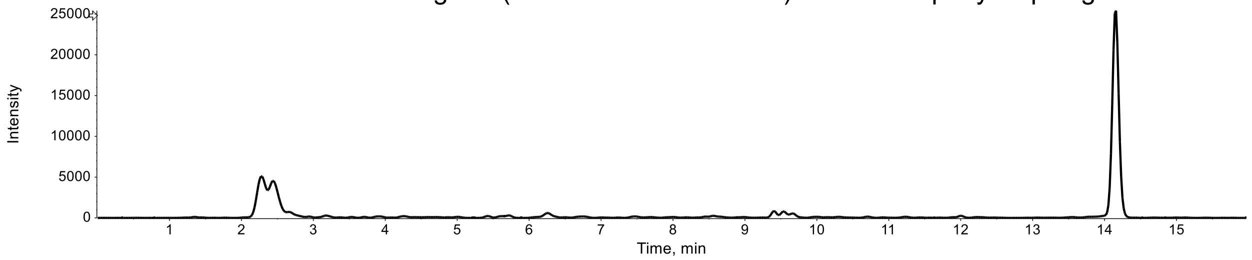

B. SWATH/MS fragment spectrum of the peak at 14.2 min.

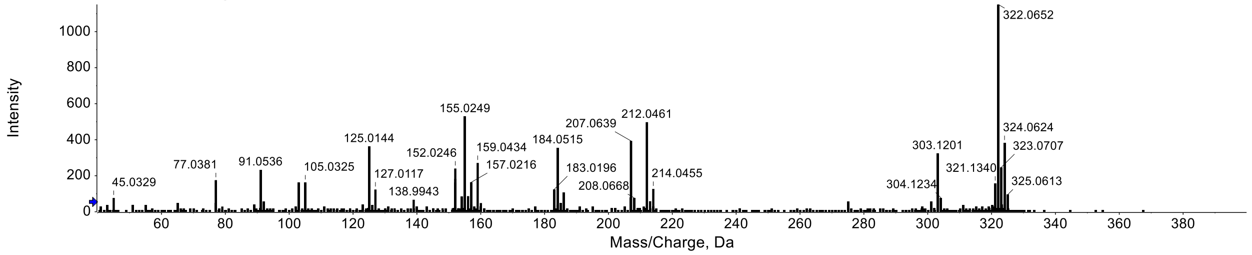

Figure S3. (**A**) MS1-level extracted ion chromatogram and (**B**) SWATH/MS fragment spectrum of clopidogrel observed in urine of a human clopidogrel user. The substance featured in this figure (presumably clopidogrel, CID 60606) reflects a ‘level 2’ identification in terms of the classification proposed by the Metabolomics Standards Initiative (Sumner LW, Amberg A, Barrett D, *et al*. Proposed minimum reporting standards for chemical analysis Chemical Analysis Working Group (CAWG) Metabolomics Standards Initiative (MSI). Metabolomics 2007; 3: 211–221).

Table S3. Overview of representative (candidate) fragments of clopidogrel (MSI level 2 identification), as depicted in Figure S3. Regarding the candidate fragments, these were derived utilizing the 'Fragment Pane' module in SCIEX PeakView (version 2.2.0.11391).

Signal	Molecular formula of candidate fragment ion	Expected m/z	Observed m/z (mass error)
Fragment 77	$C_6H_5^+$	77.0391	77.0381 (-13)
Fragment 91	$C_7H_7^+$	91.0548	91.0536 (-13)
Fragment 125	$C_7H_6Cl^+$	125.0158	125.0144 (-11)
Fragment 152	$C_8H_7ClN^+$	152.0267	152.0246 (-14)
Fragment 155	$C_8H_8ClO^+$	155.0264	155.0249 (-10)
Fragment 212	$C_{10}H_{11}ClNO_2^+$	212.0478	212.0461 (-8)
Residual Precursor	$C_{16}H_{17}ClNO_2S^+$	322.0669	322.0652 (-5)

A. MS1-level extracted ion chromatogram (m/z 304.0188 ± 0.0125) of an exemplary clopidogrel user

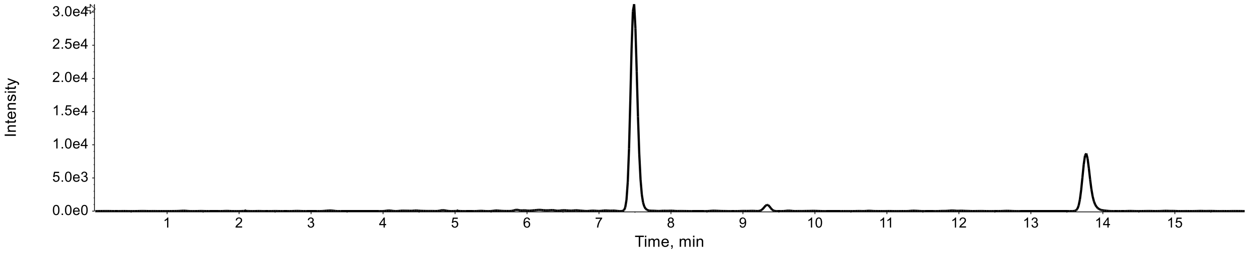

B. SWATH/MS fragment spectrum of the peak at 7.5 min.

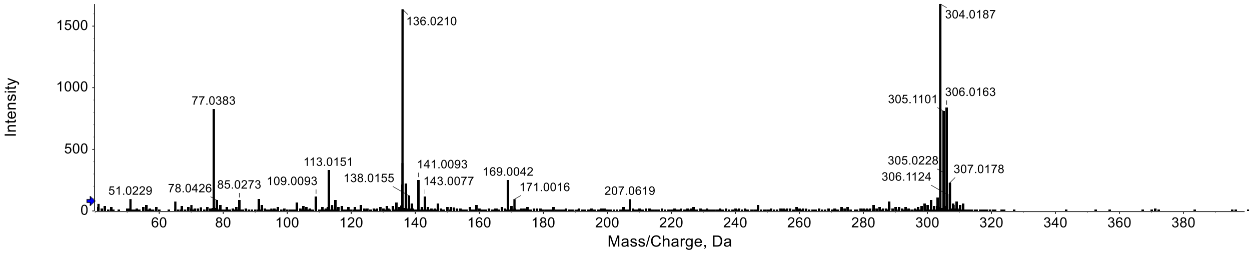

Figure S4. (**A**) MS1-level extracted ion chromatogram and (**B**) SWATH/MS fragment spectrum of a putative clopidogrel metabolite with an *m/z* value of 304.02 observed in urine of a human clopidogrel user.

Table S4. Overview of representative (candidate) fragments of the putative doubly oxidized clopidogrel carboxylic acid metabolite (M10; MSI level 3 identification), as depicted in Figure S4. Regarding the candidate fragments, these were derived utilizing the 'Fragment Pane' module in SCIEX PeakView (version 2.2.0.11391).

Signal	Molecular formula of candidate fragment ion	Expected m/z	Observed m/z (mass error)
Fragment 77	$C_6H_5^+$	77.0391	77.0383 (-10)
Fragment 141	$C_7H_6ClO^+$	141.0107	141.0093 (-10)
Fragment 169	$C_8H_6ClO_2^+$	169.0056	169.0042 (-8)
Residual Precursor	$C_{15}H_{11}ClNO_2S^+$	304.0199	304.0187 (-4)

A. MS1-level extracted ion chromatogram (m/z 306.0335 ± 0.0125) of an exemplary clopidogrel user

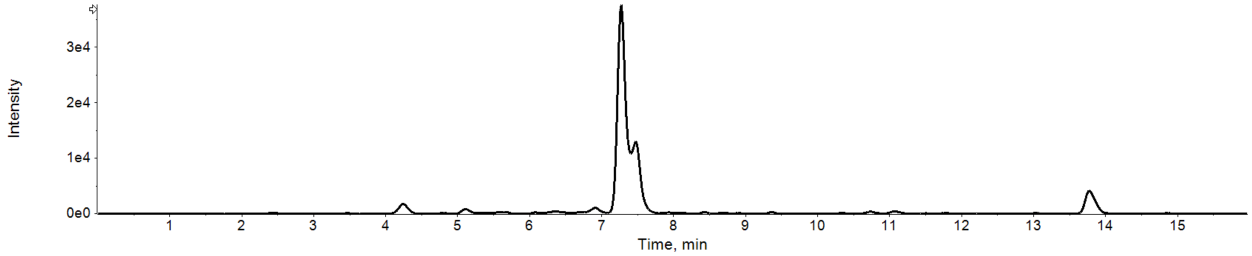

B. SWATH/MS fragment spectrum of the peak at 7.3 min.

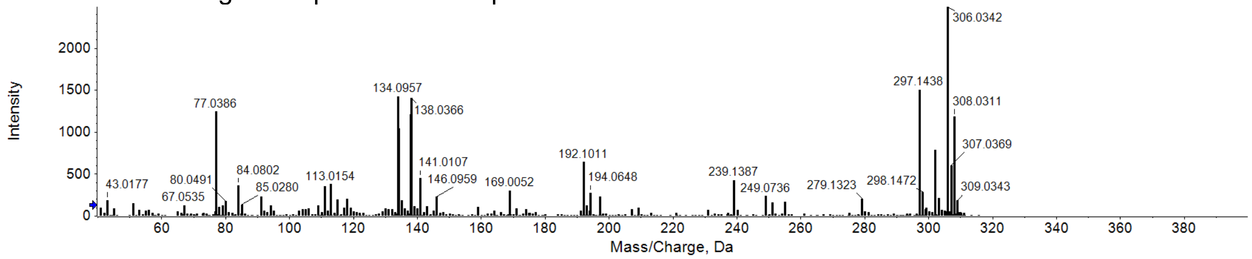

Figure S5. (**A**) MS1-level extracted ion chromatogram and (**B**) SWATH/MS fragment spectrum of a putative clopidogrel metabolite with an *m/z* value of 306.03 observed in urine of a human clopidogrel user.

A. MS1-level extracted ion chromatogram (m/z 308.0502 ± 0.0125) of an exemplary clopidogrel user

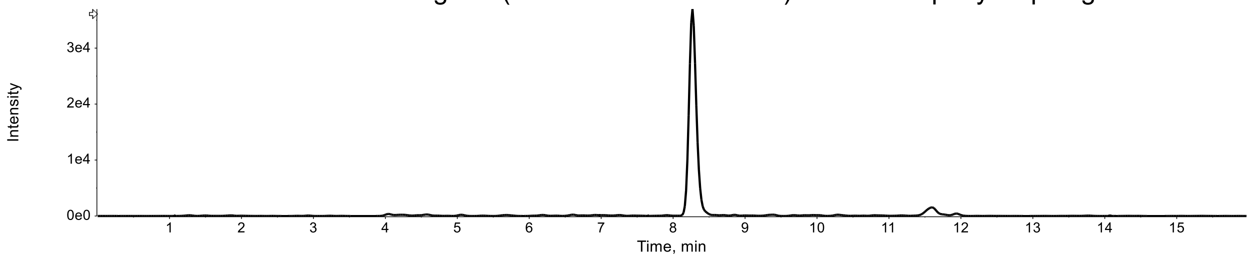

B. SWATH/MS fragment spectrum of the peak at 8.3 min.

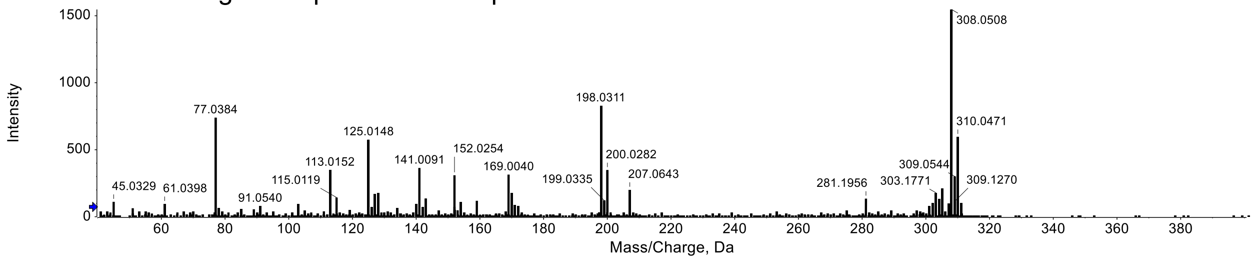

Figure S6. (**A**) MS1-level extracted ion chromatogram and (**B**) SWATH/MS fragment spectrum of a putative clopidogrel metabolite with an *m/z* value of 308.05 observed in urine of a human clopidogrel user.

Table S5. Overview of representative (candidate) fragments of the putative clopidogrel carboxylic acid metabolite (MSI level 3 identification), as depicted in Figure S6. Regarding the candidate fragments, these were derived utilizing the 'Fragment Pane' module in SCIEX PeakView (version 2.2.0.11391).

Signal	Molecular formula of candidate fragment ion	Expected m/z	Observed m/z (mass error)
Fragment 77	$C_6H_5^+$	77.0391	77.0384 (-9)
Fragment 125	$C_7H_6Cl^+$	125.0158	125.0148 (-8)
Fragment 141	$C_7H_6ClO^+$	141.0107	141.0091 (-11)
Fragment 152	$C_8H_7ClN^+$	152.0267	152.0254 (-9)
Fragment 169	$C_8H_6ClO_2^+$	169.0056	169.0040 (-9)
Fragment 198	$C_9H_9ClNO_2^+$	198.0322	198.0311 (-6)
Residual Precursor	$C_{15}H_{15}ClNO_2S^+$	308.0512	308.0508 (-1)

A. MS1-level extracted ion chromatogram (m/z 310.0830 ± 0.0125) of an exemplary clopidogrel user

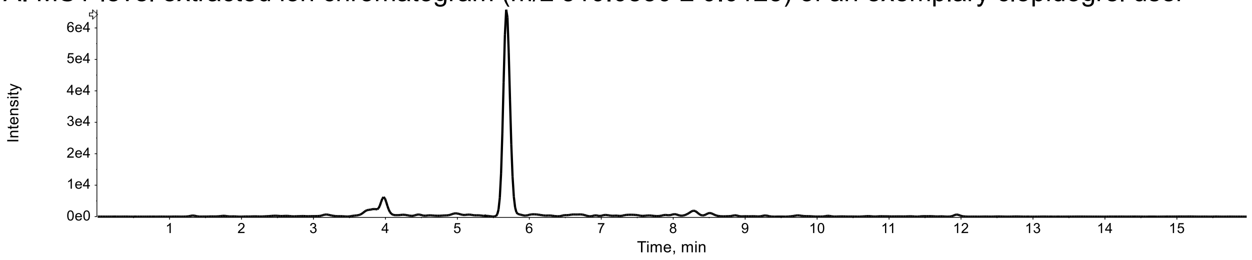

B. SWATH/MS fragment spectrum of the peak at 5.7 min.

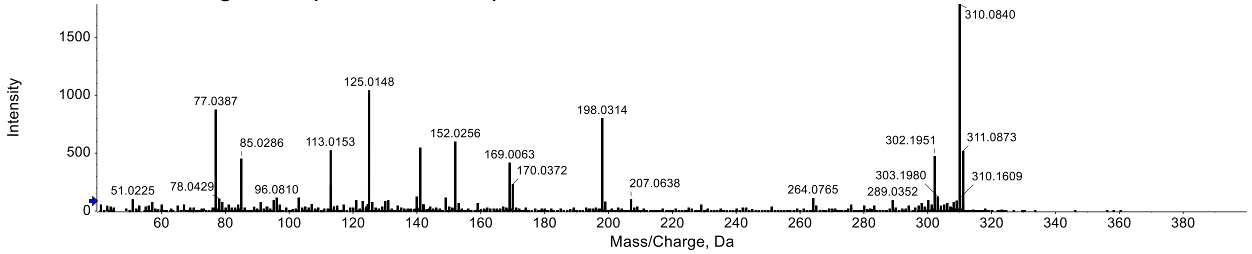

Figure S7. (**A**) MS1-level extracted ion chromatogram and (**B**) SWATH/MS fragment spectrum of a putative clopidogrel metabolite with an *m/z* value of 310.08 observed in urine of a human clopidogrel user.

A. MS1-level extracted ion chromatogram (m/z 326.0605 ± 0.0125) of an exemplary clopidogrel user

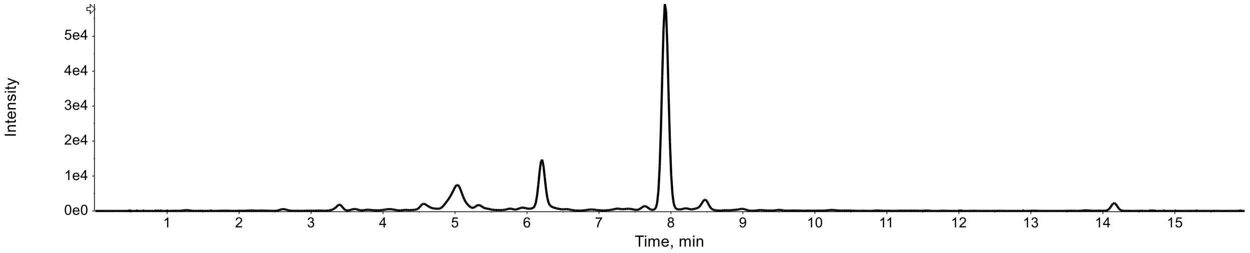

B. SWATH/MS fragment spectrum of the peak at 6.2 min.

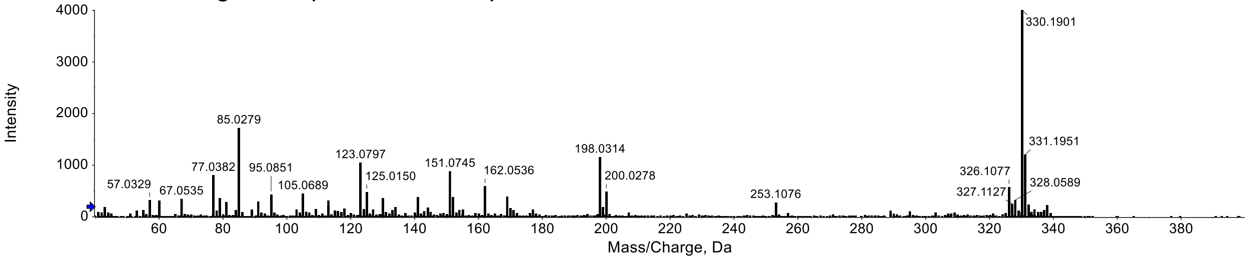

C. SWATH/MS fragment spectrum of the peak at 7.9 min.

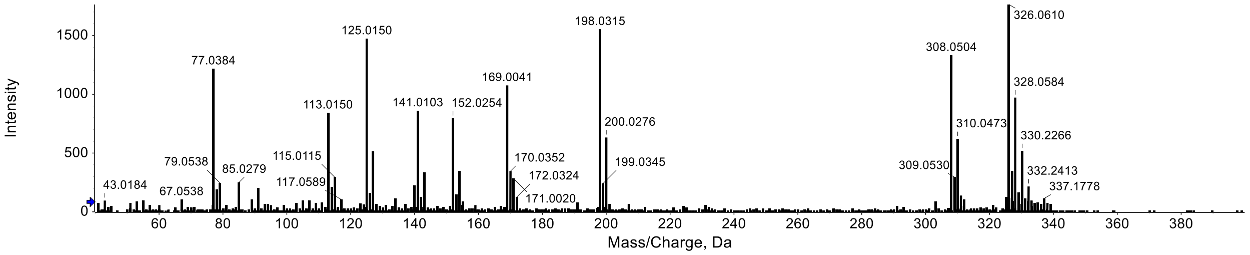

Figure S8. (**A**) MS1-level extracted ion chromatogram and (**B**-**C**) SWATH/MS fragment spectra of putative clopidogrel metabolites with an *m/z* value of 326.06 observed in urine of a human clopidogrel user.

A. MS1-level extracted ion chromatogram (m/z 328.0939 ± 0.0125) of an exemplary clopidogrel user

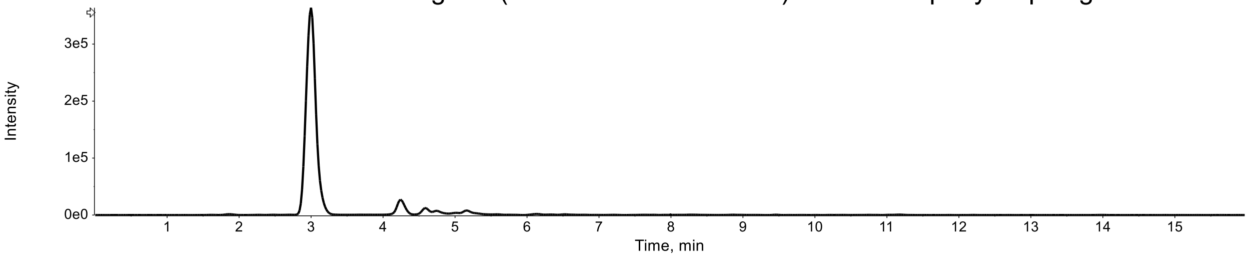

B. SWATH/MS fragment spectrum of the peak at 4.2 min.

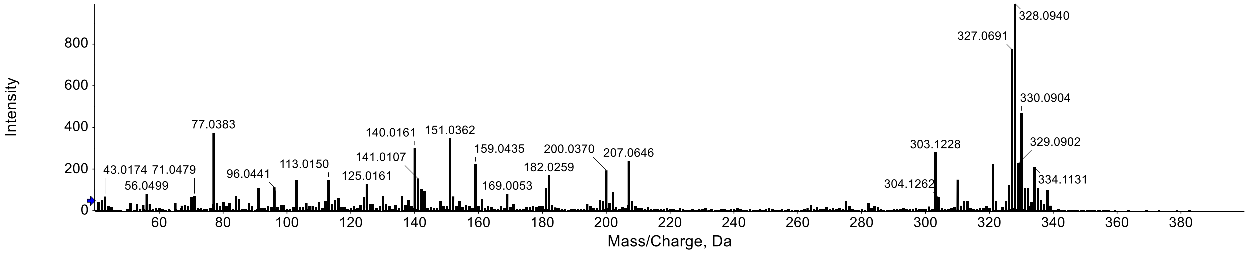

Figure S9. (**A**) MS1-level extracted ion chromatogram and (**B**) SWATH/MS fragment spectrum of a putative clopidogrel metabolite with an *m/z* value of 328.09 observed in urine of a human clopidogrel user.

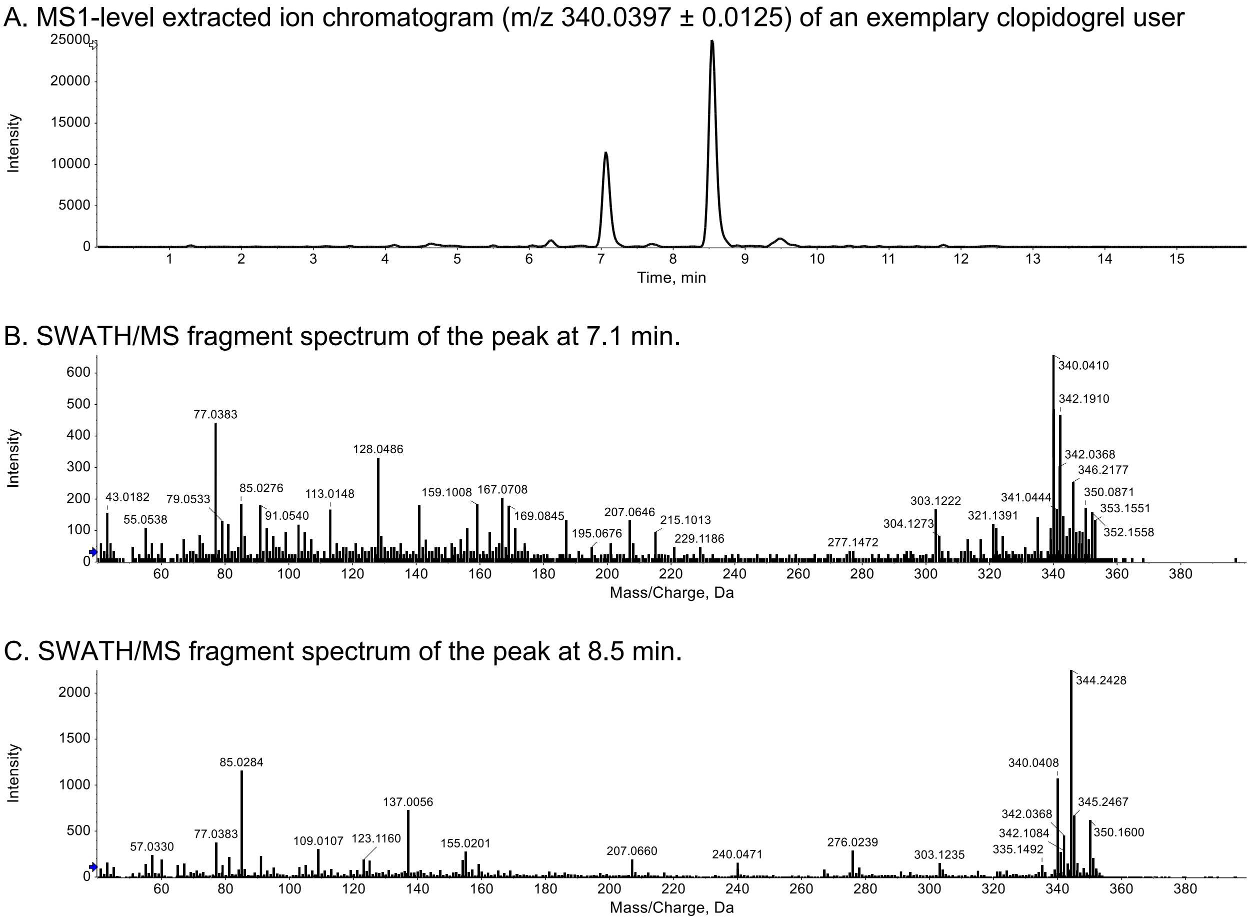

Figure S10. (**A**) MS1-level extracted ion chromatogram and (**B**-**C**) SWATH/MS fragment spectra of putative clopidogrel metabolites with an *m/z* value of 340.04 observed in urine of a human clopidogrel user.

Table S6. Overview of representative (candidate) fragments of two putative di-oxygenated clopidogrel carboxylic acid metabolites (MSI level 3 identification), as depicted in Figure S10. Regarding the candidate fragments, these were derived utilizing the 'Fragment Pane' module in SCIEX PeakView (version 2.2.0.11391).

Signal	Molecular formula of candidate fragment ion	Expected m/z	Observed m/z (mass error) at 7.1 min	Observed m/z (mass error) at 8.5 min
Fragment 77	$C_6H_5^+$	77.0391	77.0383 (-10)	77.0383 (-10)
Fragment 91	$C_7H_7^+$	91.0548	91.0540 (-9)	unlabeled
Fragment 155	$C_8H_8ClO^+$	155.0264	unlabeled	155.0201 *(-41)*
Residual Precursor	$C_{15}H_{15}ClNO_4S^+$	340.0410	340.0410 (0)	340.0408 (-1)

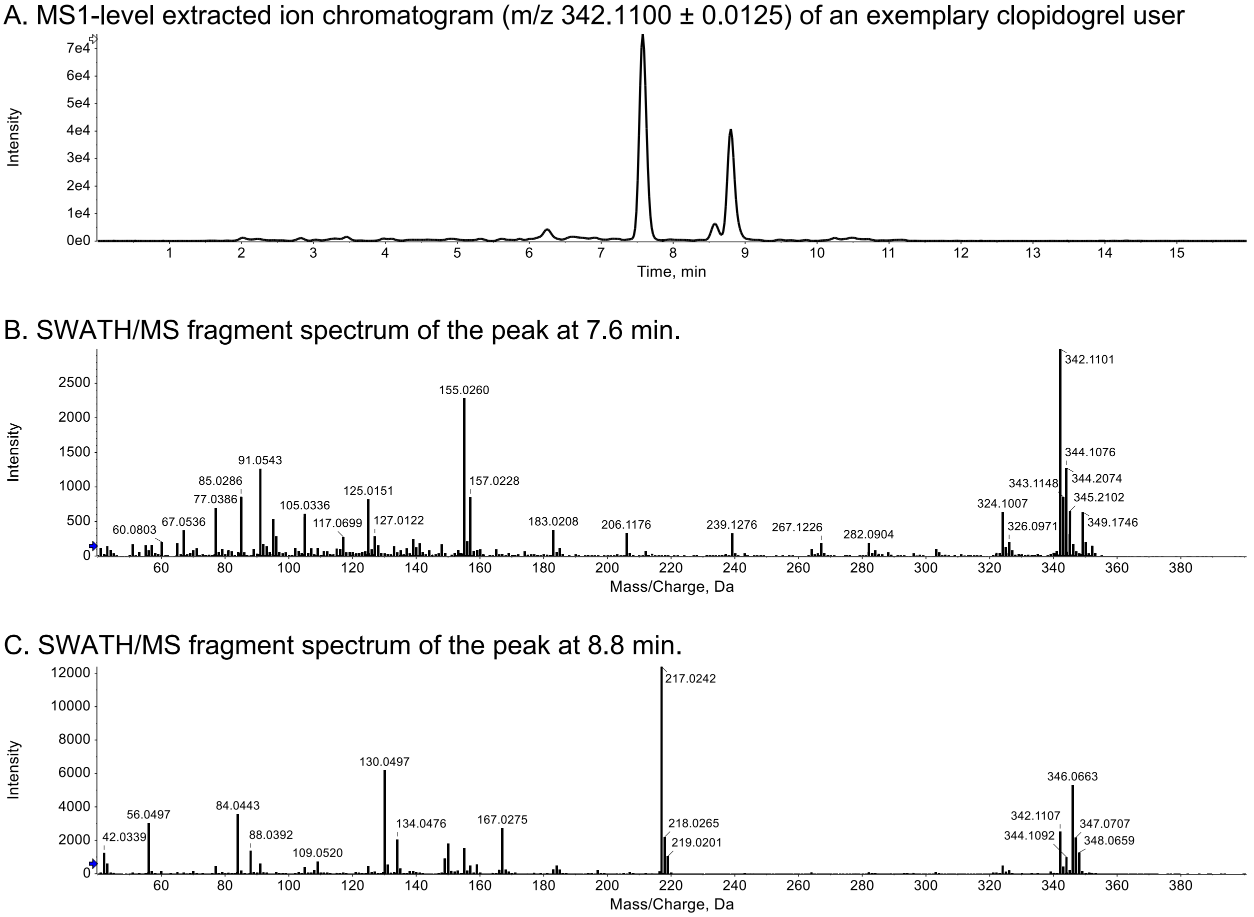

Figure S11. (**A**) MS1-level extracted ion chromatogram and (**B**-**C**) SWATH/MS fragment spectra of putative clopidogrel metabolites with an *m/z* value of 342.11 observed in urine of a human clopidogrel user.

Table S7. Overview of representative (candidate) fragments of two putative clopidogrel-thiol-derived secondary alcohol metabolites (M18H; MSI level 3 identification), as depicted in Figure S11. Regarding the candidate fragments, these were derived utilizing the 'Fragment Pane' module in SCIEX PeakView (version 2.2.0.11391).

Signal	Molecular formula of candidate fragment ion	Expected m/z	Observed m/z (mass error) at 7.6 min	Observed m/z (mass error) at 8.8 min
Fragment 77	$C_6H_5^+$	77.0391	77.0386 (-6)	unlabeled
Fragment 91	$C_7H_7^+$	91.0548	91.0543 (-5)	unlabeled
Fragment 125	$C_7H_6Cl^+$	125.0158	125.0151 (-6)	unlabeled
Fragment 155	$C_8H_8ClO^+$	155.0264	155.0260 (-3)	unlabeled
Residual Precursor	$C_{16}H_{21}ClNO_5$+	342.1108	342.1101 (-2)	342.1107 (0)

A. MS1-level extracted ion chromatogram (m/z 356.0696 ± 0.0125) of an exemplary clopidogrel user

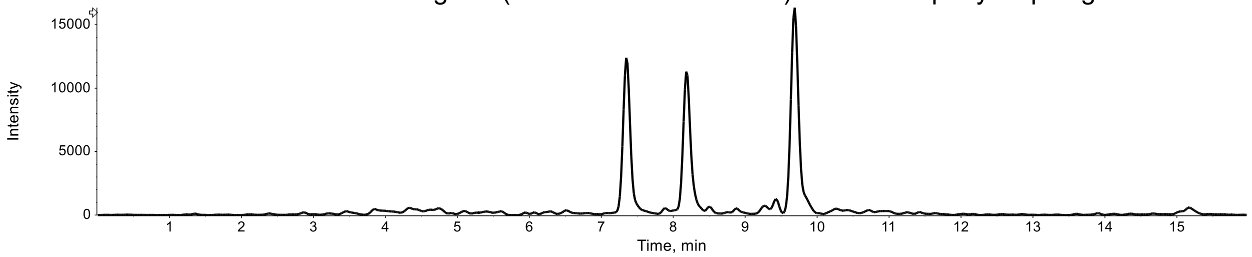

B. SWATH/MS fragment spectrum of the peak at 7.4 min.

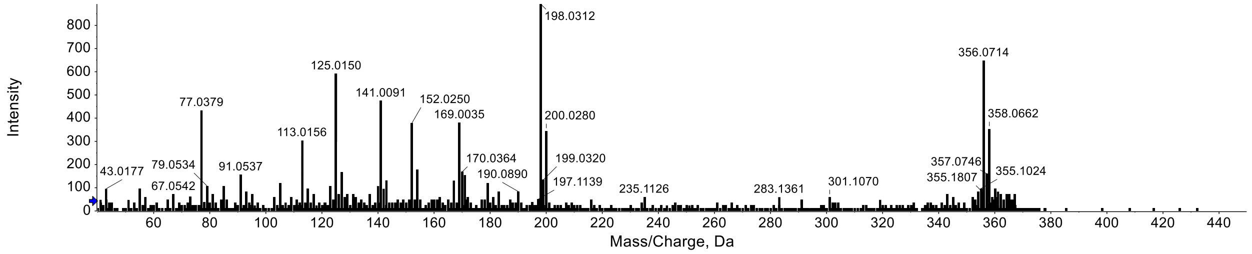

C. SWATH/MS fragment spectrum of the peak at 8.2 min.

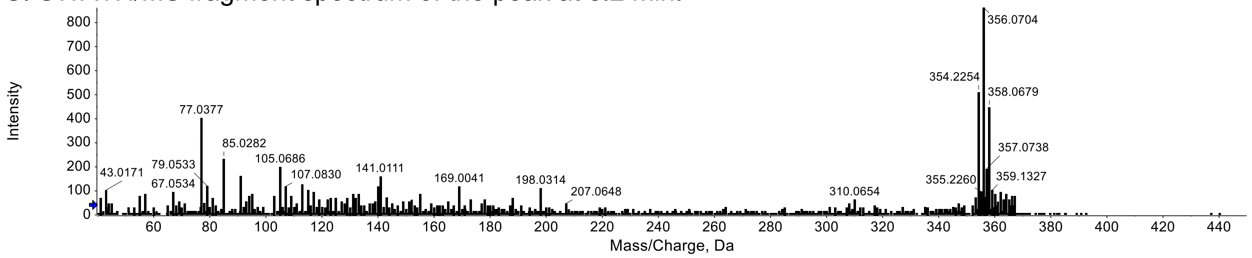

Figure S12. (**A**) MS1-level extracted ion chromatogram and (**B**-**C**) SWATH/MS fragment spectra of putative clopidogrel metabolites with an *m/z* value of 356.07 observed in urine of a human clopidogrel user.

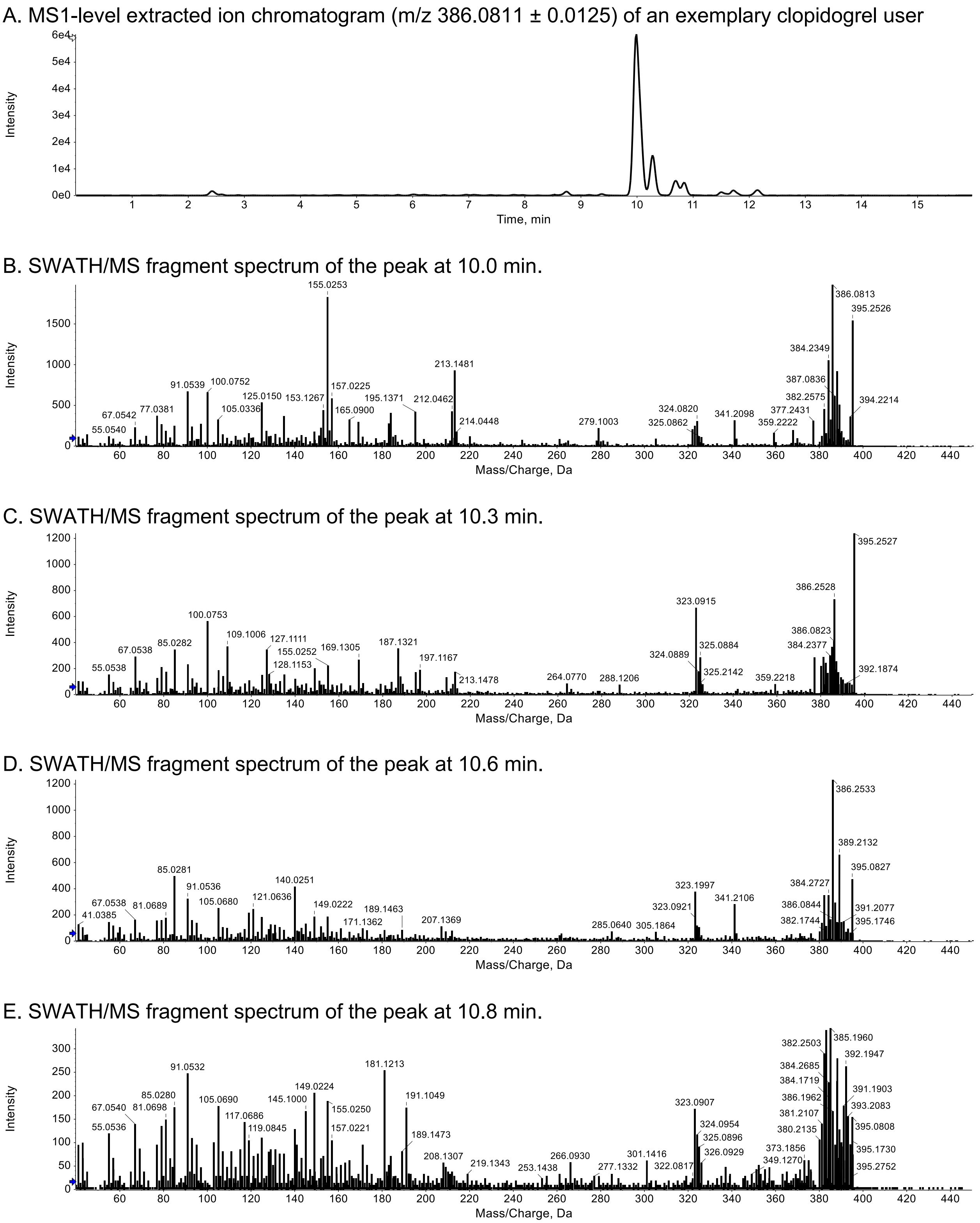

Figure S13. (**A**) MS1-level extracted ion chromatogram and (**B**-**E**) SWATH/MS fragment spectra of putative clopidogrel metabolites with an *m/z* value of 386.08 observed in urine of a human clopidogrel user.

A. MS1-level extracted ion chromatogram (m/z 427.0538 ± 0.0125) of an exemplary clopidogrel user

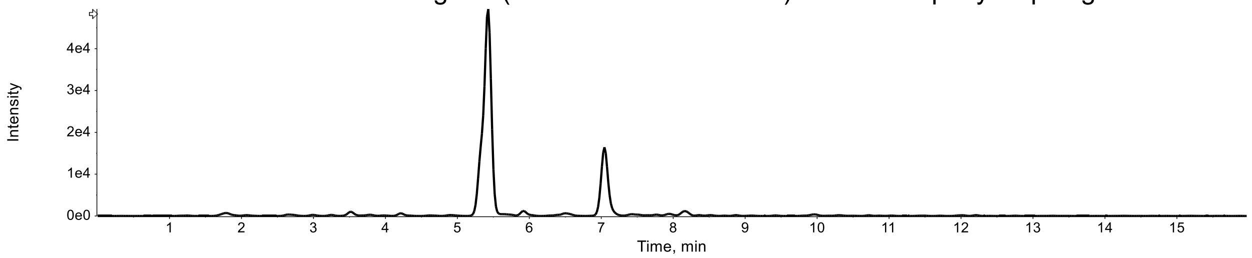

B. SWATH/MS fragment spectrum of the peak at 5.4 min.

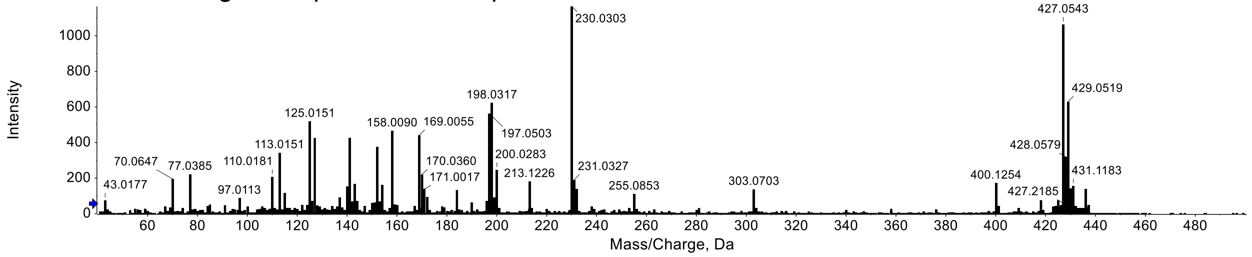

Figure S14. (**A**) MS1-level extracted ion chromatogram and (**B**) SWATH/MS fragment spectrum of a putative clopidogrel metabolite with an *m/z* value of 427.05 observed in urine of a human clopidogrel user.

A. MS1-level extracted ion chromatogram (m/z 445.0624 ± 0.0125) of an exemplary clopidogrel user

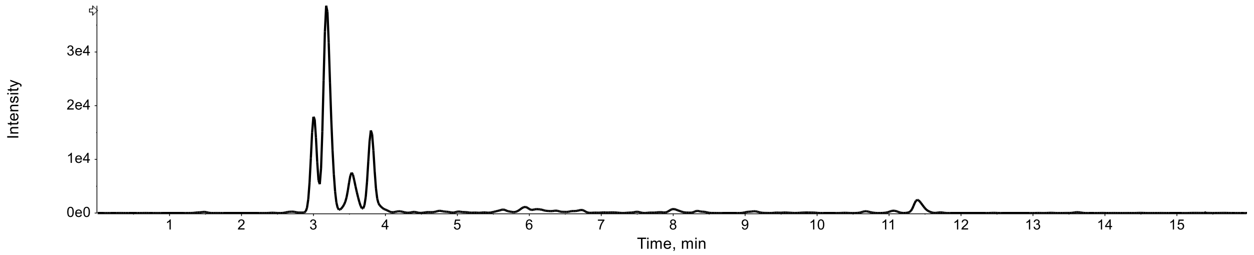

B. SWATH/MS fragment spectrum of the peak at 3.0 min.

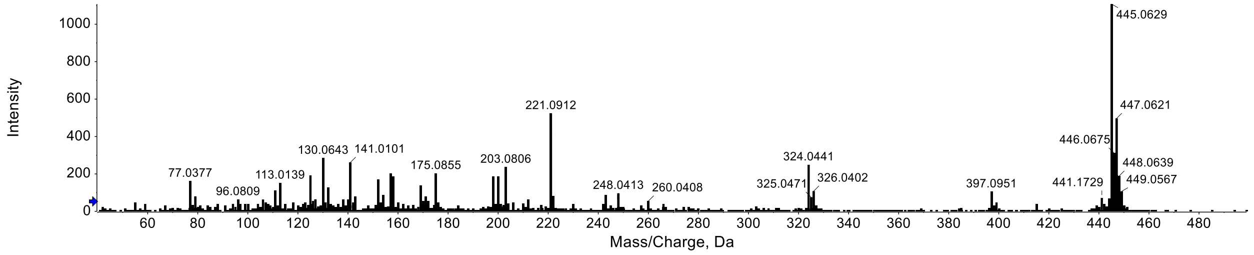

C. SWATH/MS fragment spectrum of the peak at 3.2 min.

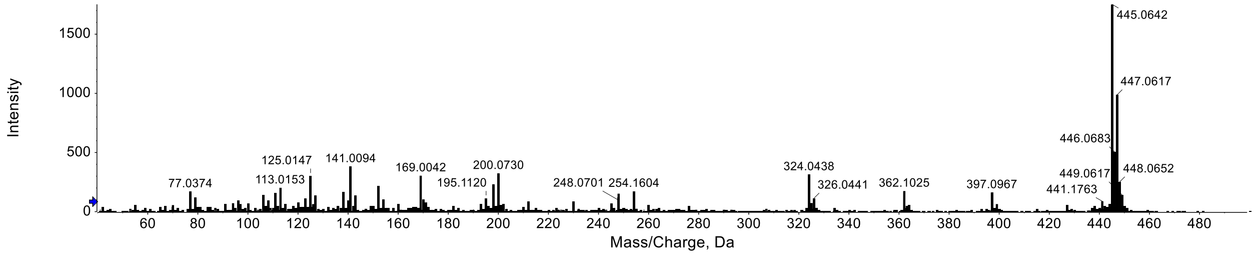

D. SWATH/MS fragment spectrum of the peak at 3.5 min.

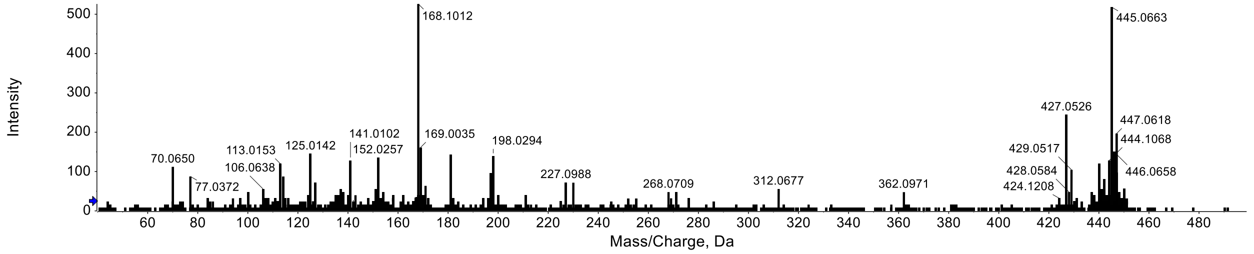

E. SWATH/MS fragment spectrum of the peak at 3.8 min.

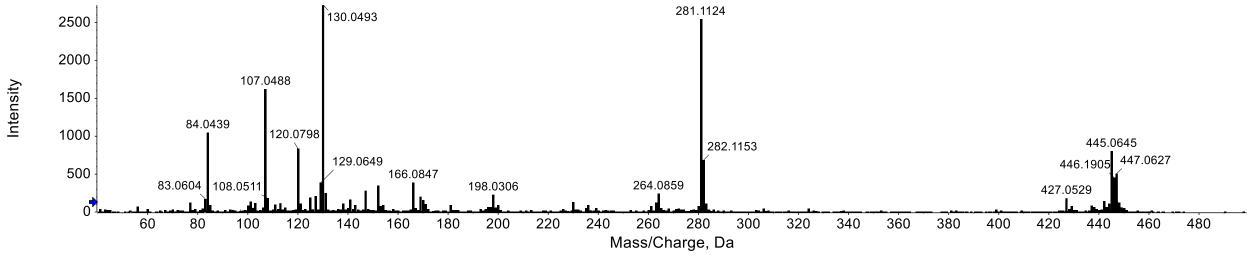

Figure S15. (**A**) MS1-level extracted ion chromatogram and (**B**-**E**) SWATH/MS fragment spectra of putative clopidogrel metabolites with an *m/z* value of 445.06 observed in urine of a human clopidogrel user.

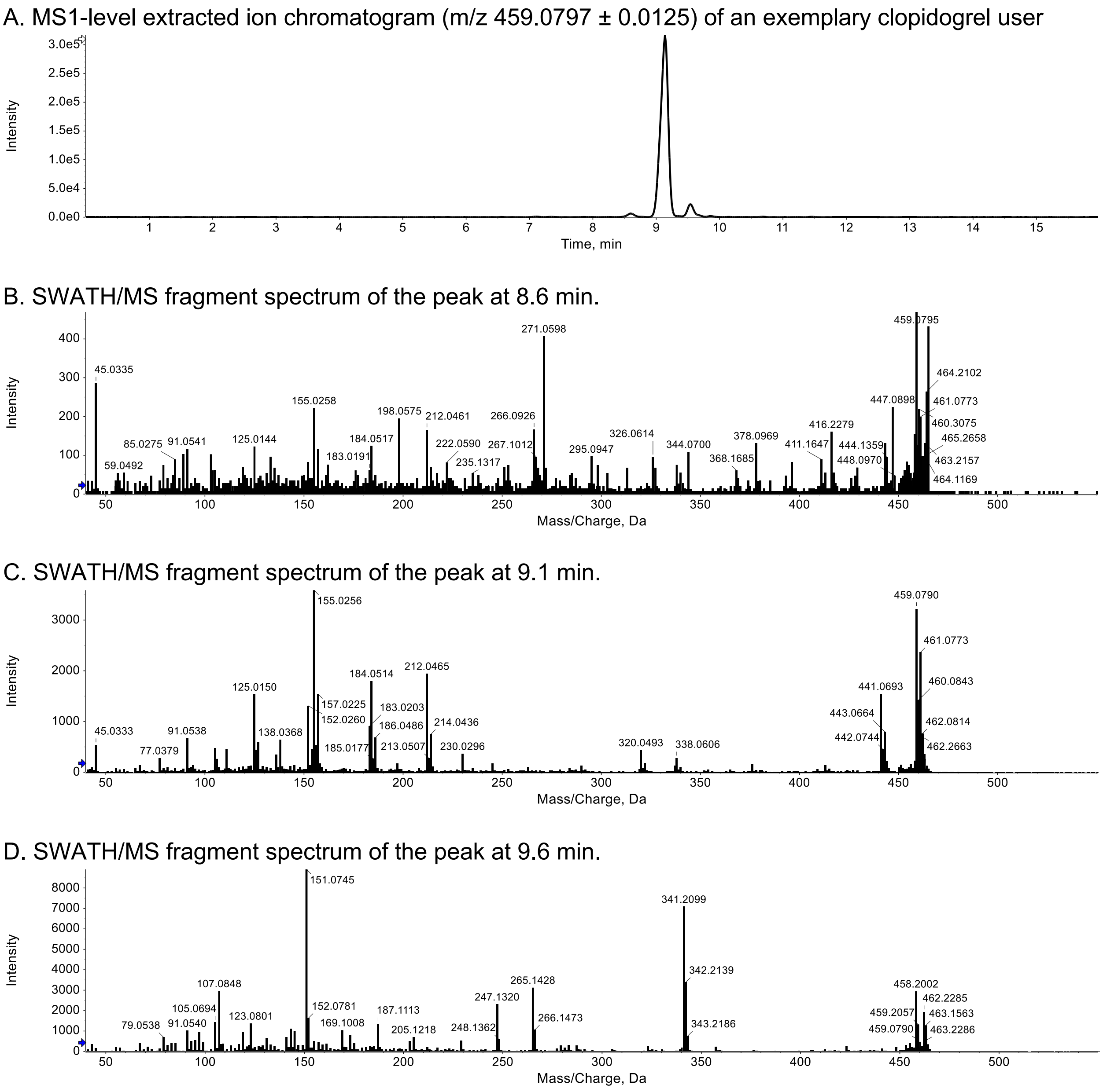

Figure S16. (**A**) MS1-level extracted ion chromatogram and (**B**-**D**) SWATH/MS fragment spectra of putative clopidogrel metabolites with an *m/z* value of 459.08 observed in urine of a human clopidogrel user.

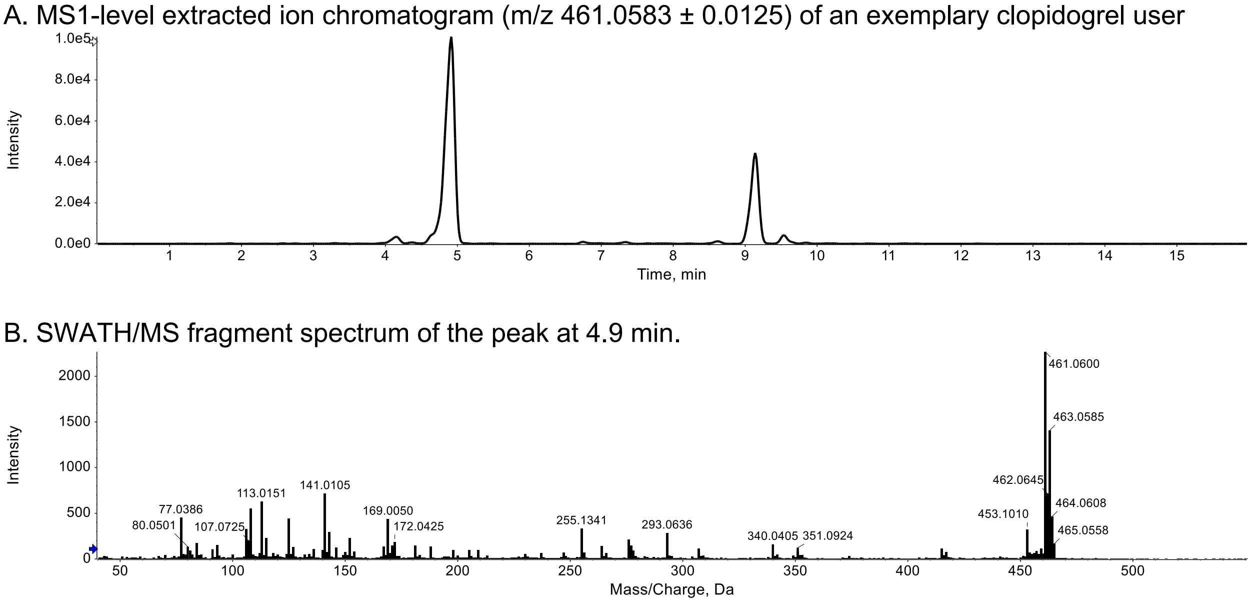

Figure S17. (**A**) MS1-level extracted ion chromatogram and (**B**) SWATH/MS fragment spectrum of a putative clopidogrel metabolite with an *m/z* value of 461.06 observed in urine of a human clopidogrel user.

A. MS1-level extracted ion chromatogram (m/z 484.0806 ± 0.0125) of an exemplary clopidogrel user

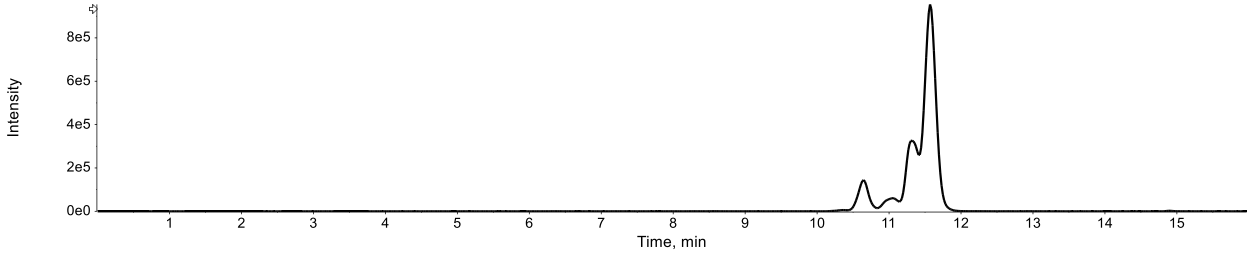

B. SWATH/MS fragment spectrum of the peak at 10.6 min.

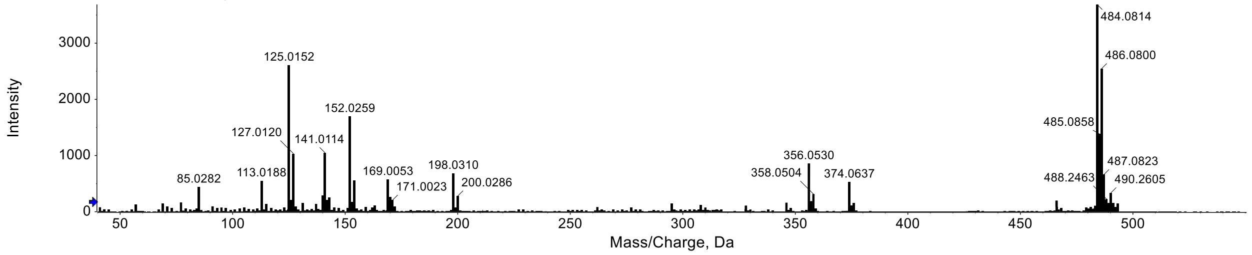

C. SWATH/MS fragment spectrum of the peak at 11.0 min.

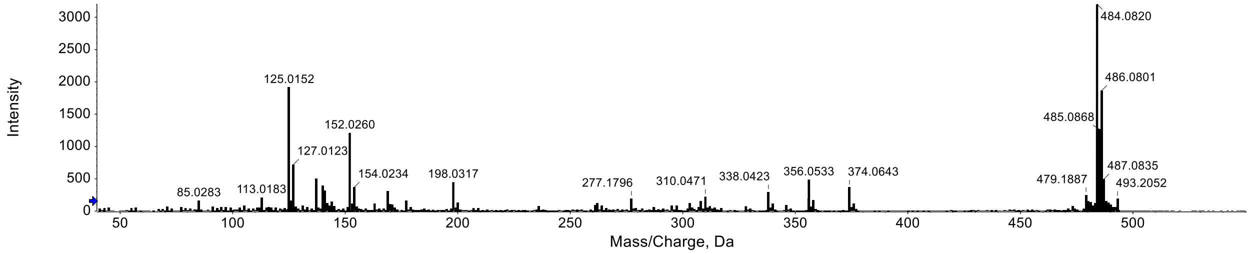

D. SWATH/MS fragment spectrum of the peak at 11.3 min.

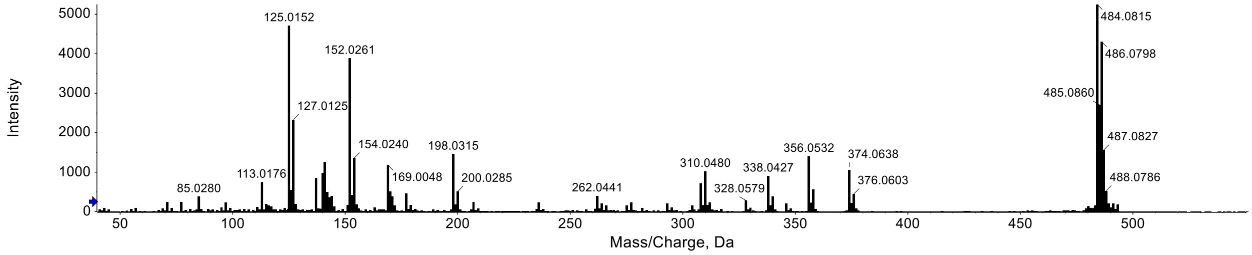

E. SWATH/MS fragment spectrum of the peak at 11.6 min.

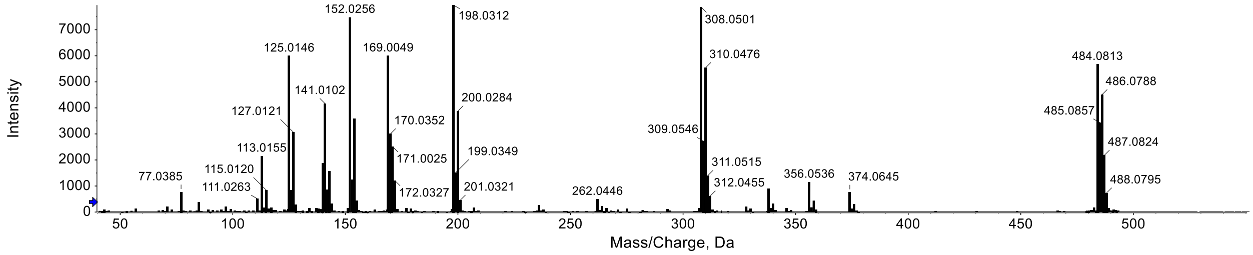

Figure S18. (**A**) MS1-level extracted ion chromatogram and (**B**-**E**) SWATH/MS fragment spectra of putative clopidogrel metabolites with an *m/z* value of 484.08 observed in urine of a human clopidogrel user.

Table S8. Overview of representative (candidate) fragments of four putative clopidogrel carboxylic acid glucuronide metabolites (MSI level 3 identification), as depicted in Figure S18. Regarding the candidate fragments, these were derived utilizing the 'Fragment Pane' module in SCIEX PeakView (version 2.2.0.11391).

Signal	Molecular formula of candidate fragment ion	Expected m/z	Observed m/z (mass error) at 10.6 min	Observed m/z (mass error) at 11.0 min	Observed m/z (mass error) at 11.3 min	Observed m/z (mass error) at 11.6 min
Fragment 125	$C_7H_6Cl^+$	125.0158	125.0152 (-5)	125.0152 (-5)	125.0161 (2)	125.0146 (-10)
Fragment 141	$C_7H_6ClO^+$	141.0107	141.0114 (5)	unlabeled	unlabeled	141.0102 (-4)
Fragment 152	$C_8H_7ClN^+$	152.0267	152.0259 (-5)	152.0260 (-5)	152.0261 (-4)	152.0256 (-7)
Fragment 169	$C_8H_6ClO_2^+$	169.0056	169.0053 (-2)	unlabeled	169.0048 (-5)	169.0049 (-4)
Fragment 198	$C_9H_9ClNO_2^+$	198.0322	198.0310 (-6)	198.0317 (-3)	198.0315 (-4)	198.0312 (-5)
Fragment 308	$C_{15}H_{15}ClNO_2S^+$	308.0512	unlabeled	unlabeled	unlabeled	308.0501 (-4)
Residual Precursor	$C_{21}H_{23}ClNO_8S^+$	484.0833	484.0814 (-4)	484.0820 (-3)	484.0815 (-4)	484.0813 (-4)

A. MS1-level extracted ion chromatogram (m/z 500.0760 ± 0.0125) of an exemplary clopidogrel user

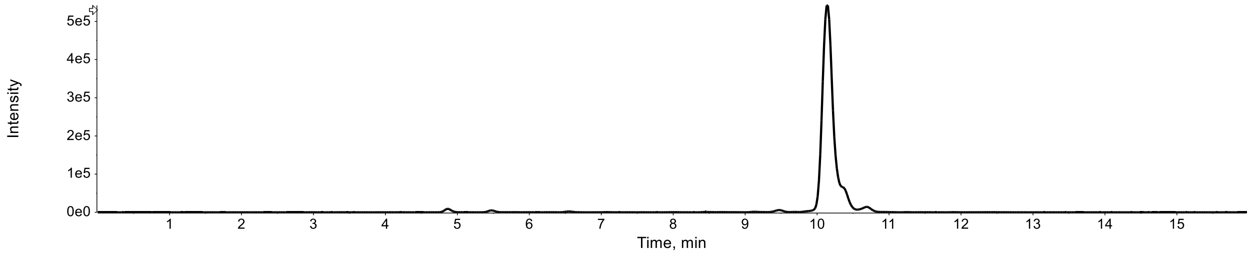

B. SWATH/MS fragment spectrum of the peak at 10.2 min.

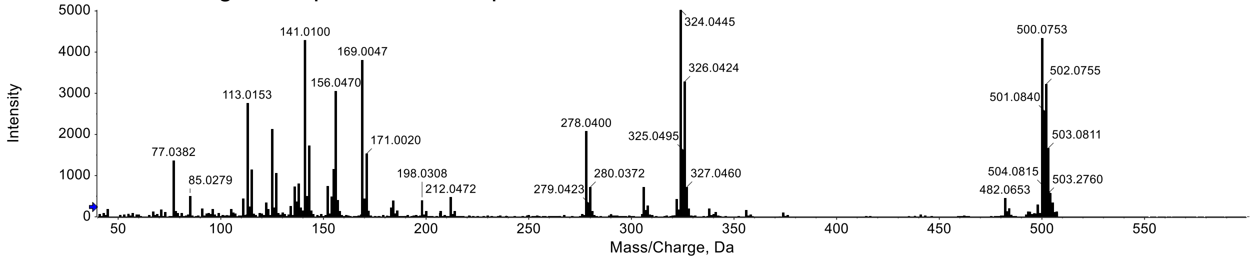

Figure S19. (**A**) MS1-level extracted ion chromatogram and (**B**) SWATH/MS fragment spectrum of a putative clopidogrel metabolite with an *m/z* value of 500.08 observed in urine of a human clopidogrel user.

Table S9. Overview of representative (candidate) fragments of the putative mono-oxygenated clopidogrel carboxylic acid glucuronide metabolite (MSI level 3 identification), as depicted in Figure S19. Regarding the candidate fragments, these were derived utilizing the 'Fragment Pane' module in SCIEX PeakView (version 2.2.0.11391).

Signal	Molecular formula of candidate fragment ion	Expected m/z	Observed m/z (mass error)
Fragment 77	$C_6H_5^+$	77.0391	77.0382 (-12)
Fragment 141	$C_7H_6ClO^+$	141.0107	141.0100 (-5)
Fragment 169	$C_8H_6ClO_2^+$	169.0056	169.0047 (-5)
Fragment 198	$C_9H_9ClNO_2^+$	198.0322	198.0308 (-7)
Fragment 212	$C_{10}H_{11}ClNO_2^+$	212.0478	212.0472 (-3)
Fragment 324	$C_{15}H_{15}ClNO_3S^+$	324.0461	324.0445 (-5)
Residual Precursor	$C_{21}H_{23}ClNO_9S^+$	500.0782	500.0753 (-6)

A. MS1-level extracted ion chromatogram (m/z 502.1082 ± 0.0125) of an exemplary clopidogrel user

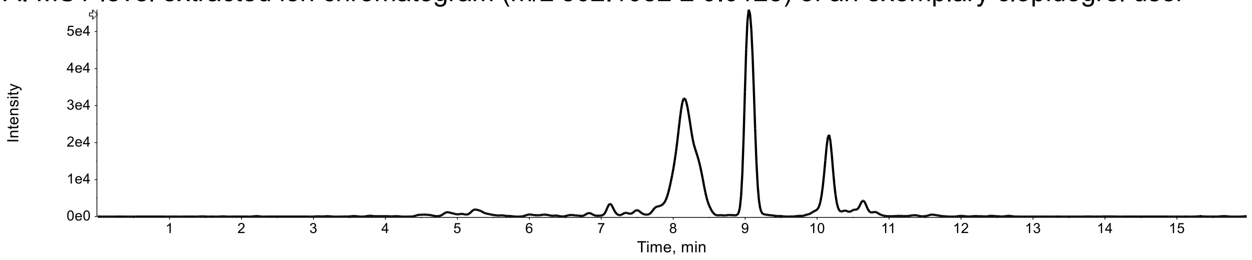

B. SWATH/MS fragment spectrum of the peak at 8.2 min.

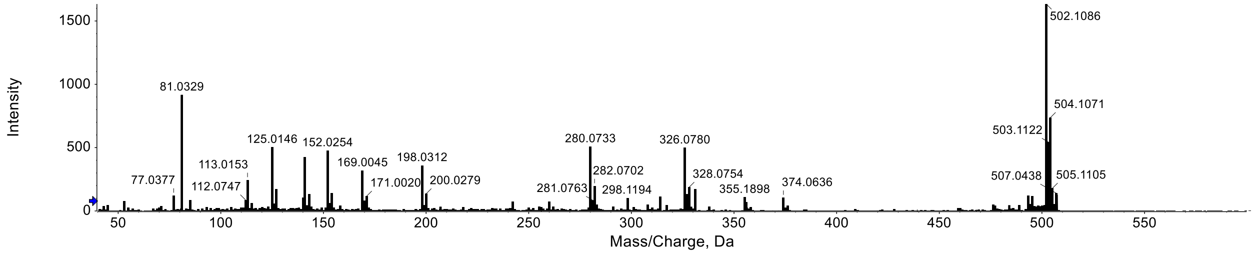

Figure S20. (**A**) MS1-level extracted ion chromatogram and (**B**) SWATH/MS fragment spectrum of a putative clopidogrel metabolite with an *m/z* value of 502.11 observed in urine of a human clopidogrel user.

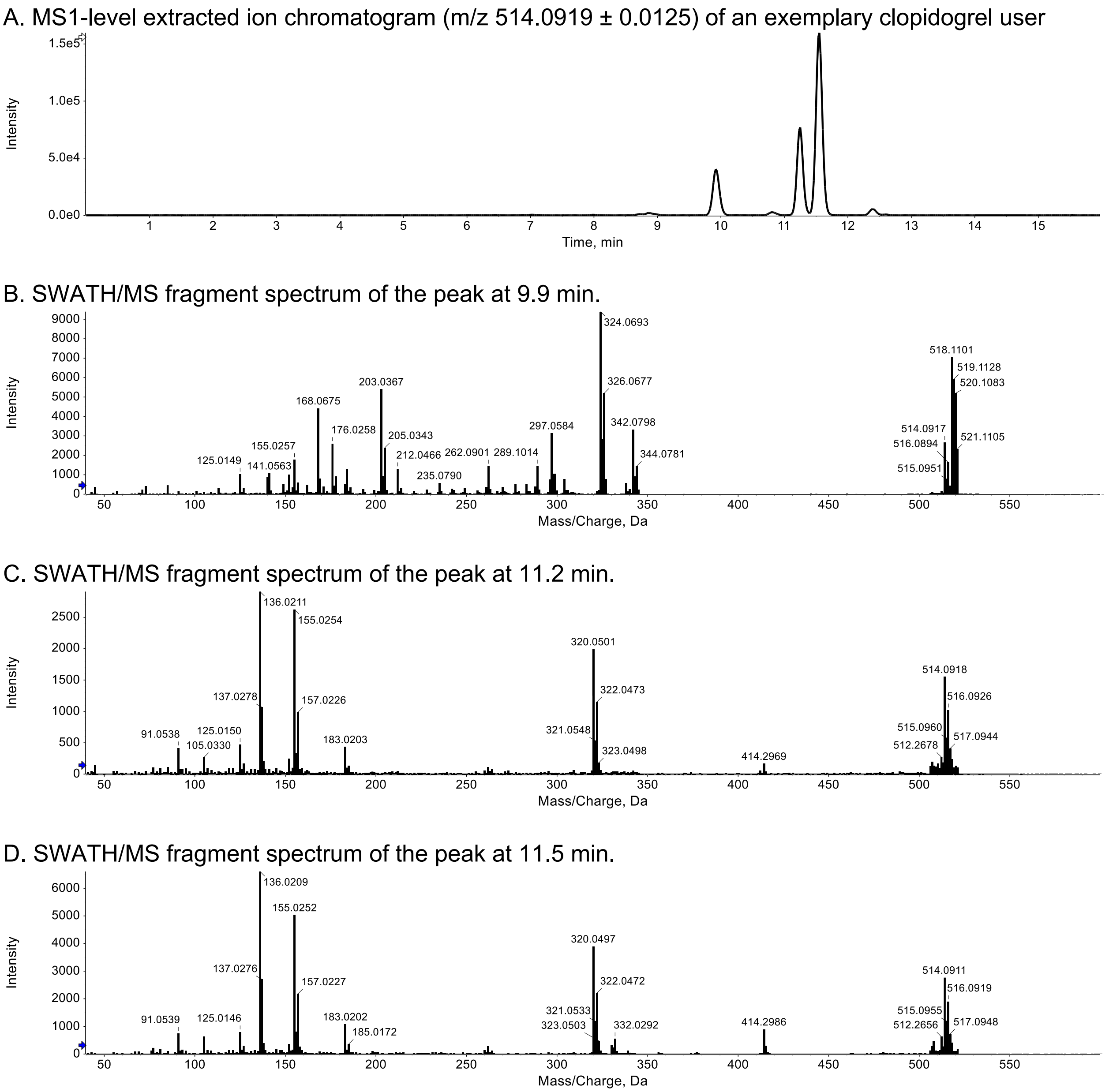

Figure S21. (**A**) MS1-level extracted ion chromatogram and (**B**-**D**) SWATH/MS fragment spectra of putative clopidogrel metabolites with an *m/z* value of 514.09 observed in urine of a human clopidogrel user.

A. MS1-level extracted ion chromatogram (m/z 516.0713 ± 0.0125) of an exemplary clopidogrel user

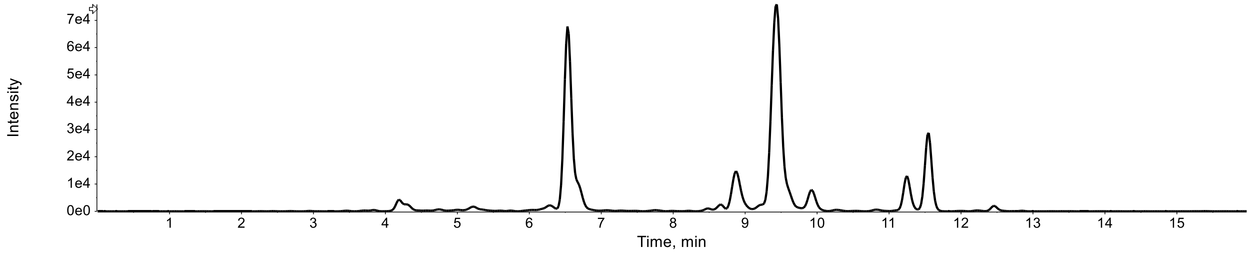

B. SWATH/MS fragment spectrum of the peak at 6.6 min.

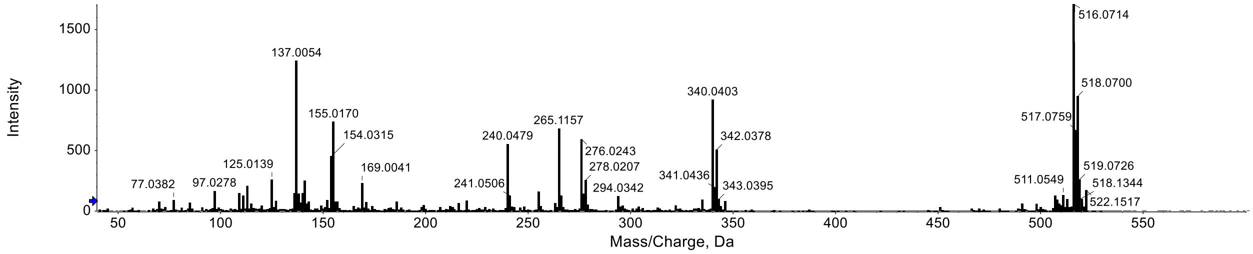

C. SWATH/MS fragment spectrum of the peak at 9.4 min.

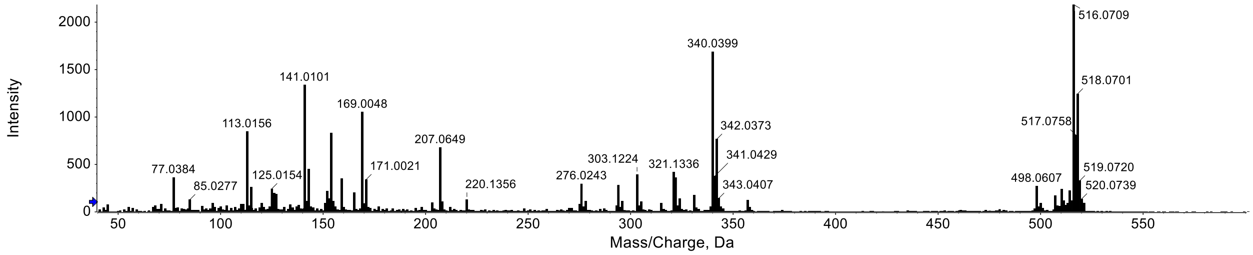

Figure S22. (**A**) MS1-level extracted ion chromatogram and (**B**-**C**) SWATH/MS fragment spectra of putative clopidogrel metabolites with an *m/z* value of 516.07 observed in urine of a human clopidogrel user.

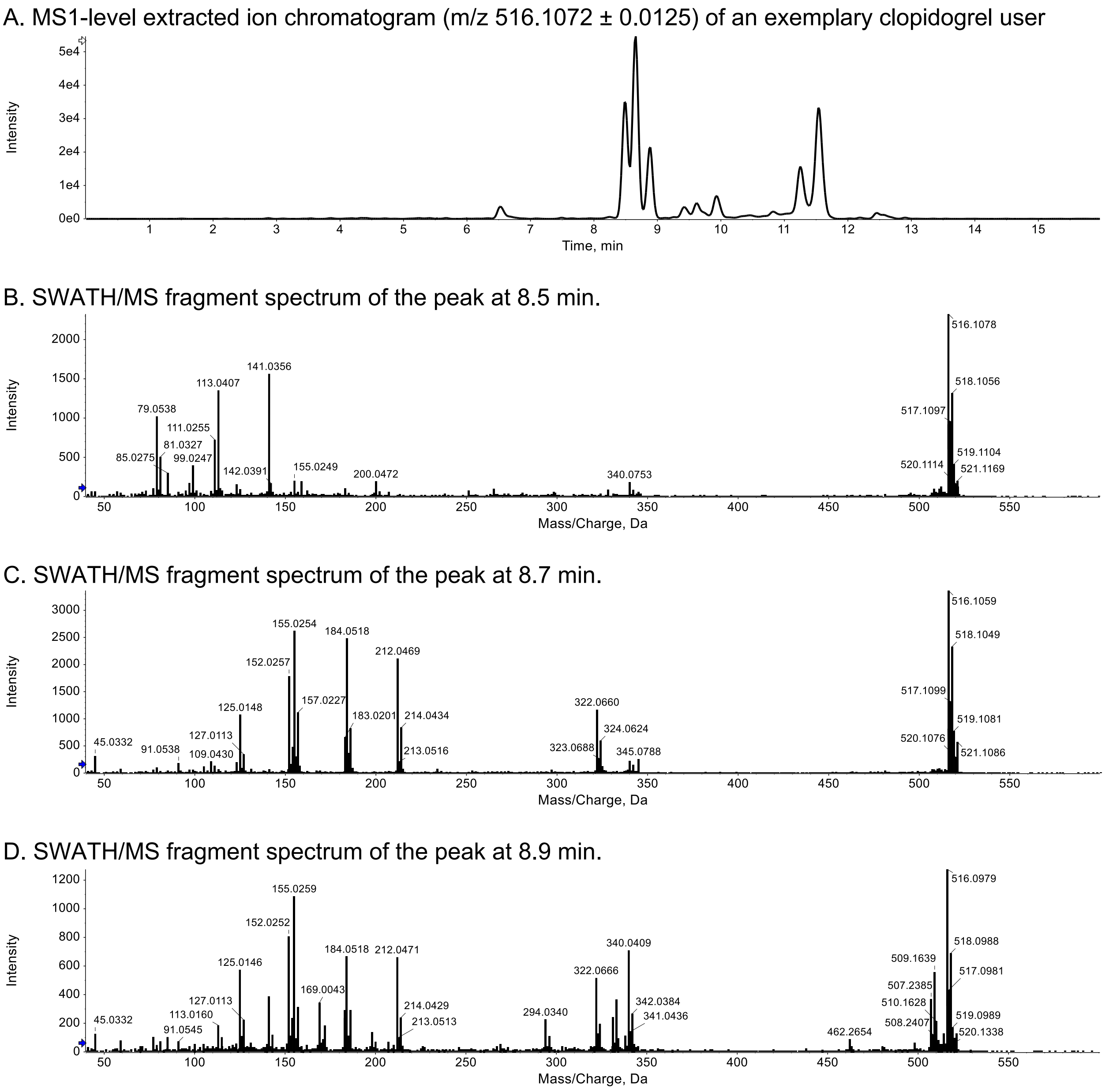

Figure S23. (**A**) MS1-level extracted ion chromatogram and (**B**-**D**) SWATH/MS fragment spectra of putative clopidogrel metabolites with an *m/z* value of 516.11 observed in urine of a human clopidogrel user.

A. MS1-level extracted ion chromatogram (m/z 518.1389 ± 0.0125) of an exemplary clopidogrel user

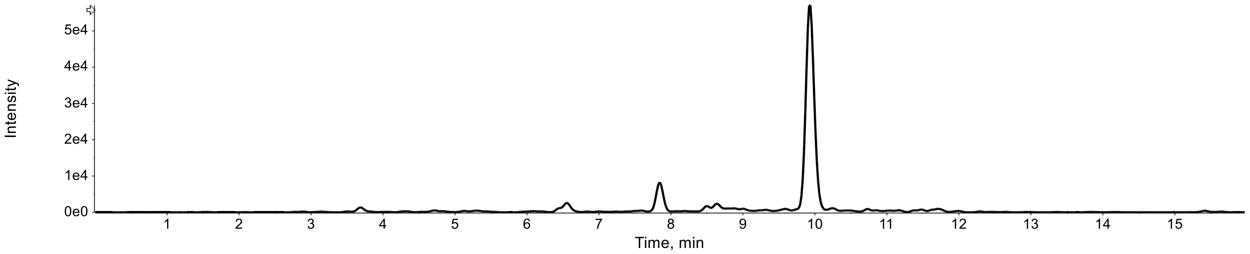

B. SWATH/MS fragment spectrum of the peak at 7.9 min.

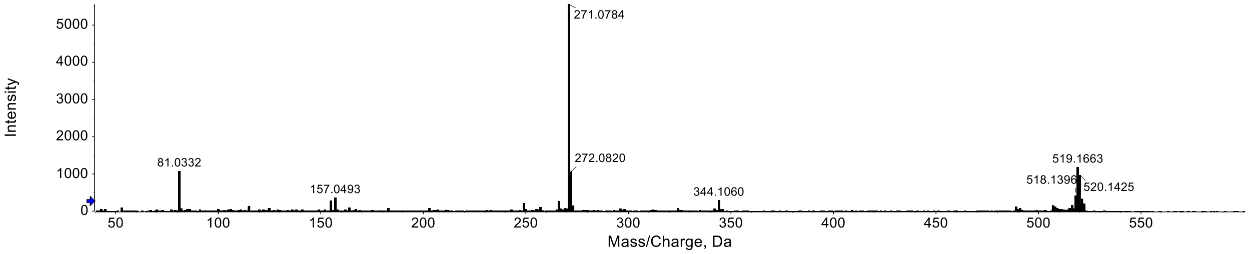

Figure S24. (**A**) MS1-level extracted ion chromatogram and (**B**) SWATH/MS fragment spectrum of a putative clopidogrel metabolite with an *m/z* value of 518.14 observed in urine of a human clopidogrel user.

A. MS1-level extracted ion chromatogram (m/z 530.0848 ± 0.0125) of an exemplary clopidogrel user

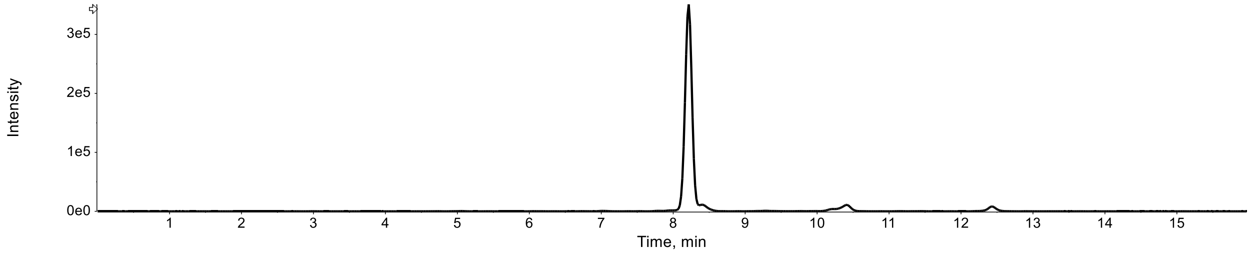

B. SWATH/MS fragment spectrum of the peak at 8.2 min.

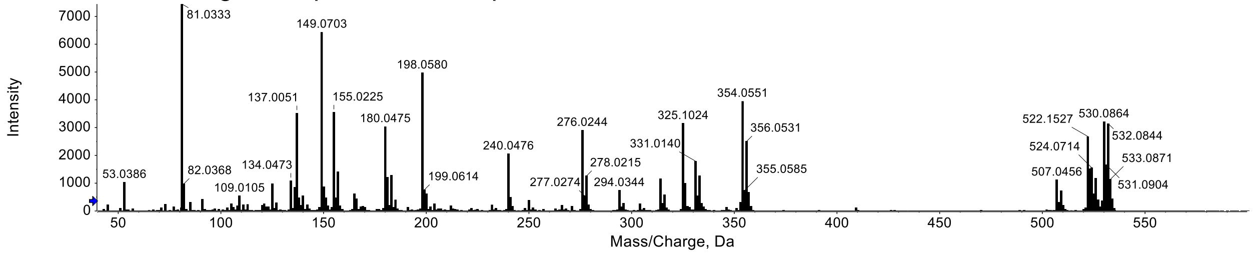

Figure S25. (**A**) MS1-level extracted ion chromatogram and (**B**) SWATH/MS fragment spectrum of a putative clopidogrel metabolite with an *m/z* value of 530.08 observed in urine of a human clopidogrel user.

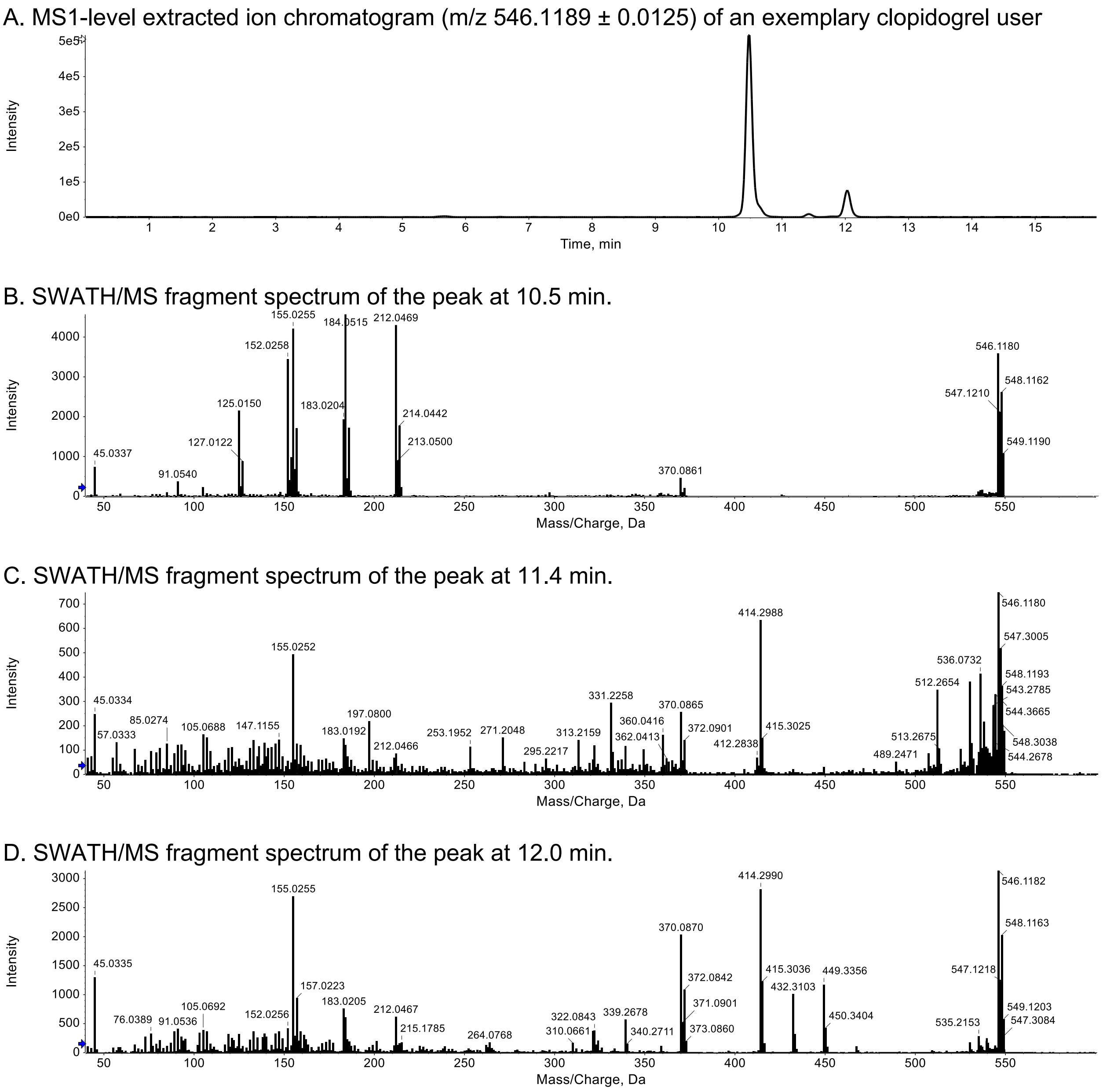

Figure S26. (**A**) MS1-level extracted ion chromatogram and (**B**-**D**) SWATH/MS fragment spectra of putative clopidogrel metabolites with an *m/z* value of 546.12 observed in urine of a human clopidogrel user.

A. MS1-level extracted ion chromatogram (m/z 637.0896 ± 0.0125) of an exemplary clopidogrel user

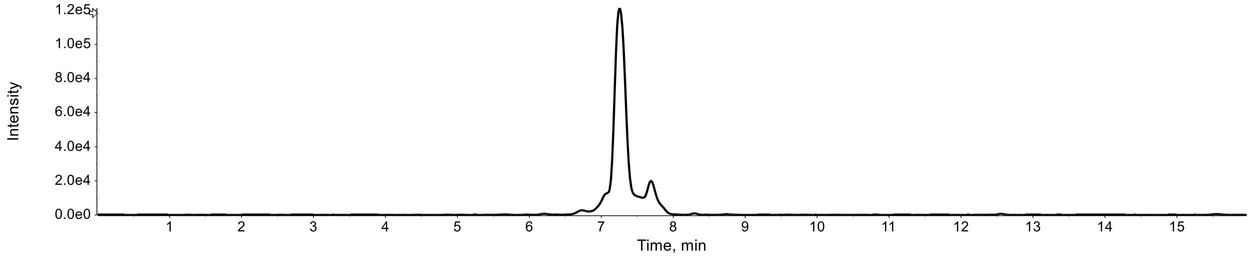

B. SWATH/MS fragment spectrum of the peak at 7.3 min.

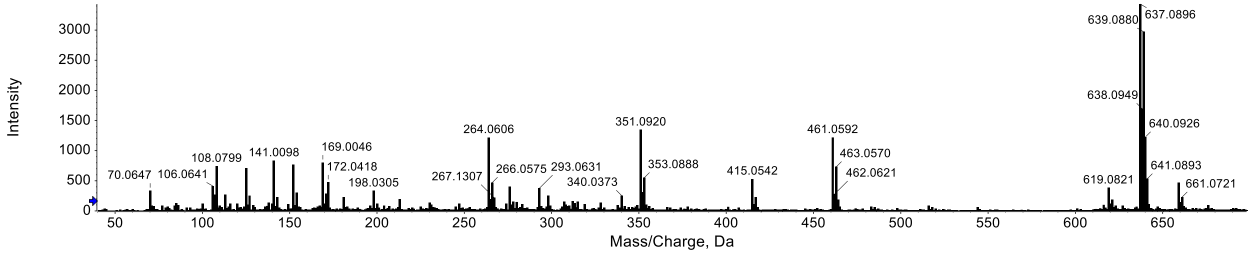

Figure S27. (**A**) MS1-level extracted ion chromatogram and (**B**) SWATH/MS fragment spectrum of a putative clopidogrel metabolite with an *m/z* value of 637.09 observed in urine of a human clopidogrel user.

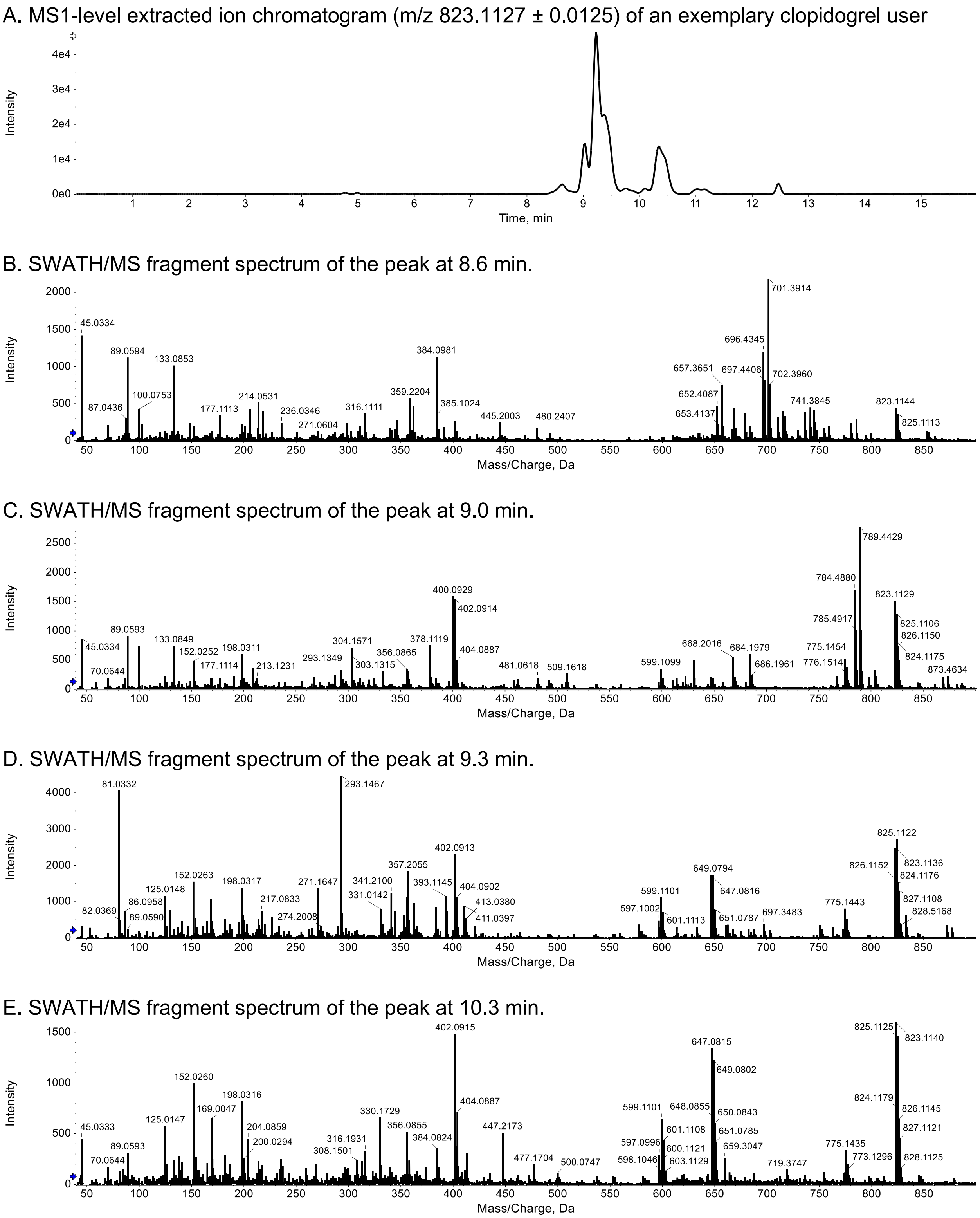

Figure S28. (**A**) MS1-level extracted ion chromatogram and (**B**-**E**) SWATH/MS fragment spectra of putative clopidogrel metabolites with an *m/z* value of 823.11 observed in urine of a human clopidogrel user.
